# A virulent bacterial signature is associated with the development of recurrence following colorectal cancer surgery

**DOI:** 10.1038/s41467-026-74889-x

**Published:** 2026-07-06

**Authors:** Meejeon Roh, Bidisha Barat, Jack A. Gilbert, Theodore Karrison, Mohsen Rouhani Ravari, Claire Wild, Nicholas R. Suss, Sara Gaines, Ryan Morgan, Kristina Martinez-Guryn, Anthony M. Martini, Olga Zaborina, Orlando DeLeon, Benjamin D. Shogan

**Affiliations:** 1 Department of Surgery, University of Chicago Medicine, Chicago, IL USA; 2 Department of Pediatrics, Center for Microbiome Innovation, University of California San Diego, La Jolla, CA USA; 3 Department of Public Health Sciences, University of Chicago Medicine, Chicago, IL USA; 4 Department of Biomedical Sciences, College of Graduate Studies, Midwestern University, Downers Grove, IL USA; 5 Laboratory of Molecular Biology, Center for Cancer Research, National Cancer Institute, National Institutes of Health, Bethesda, MD USA; 6 Department of Medicine, University of Chicago Medicine, Chicago, IL USA; 7 Chan Zuckerberg Biohub, Chicago, IL USA

**Keywords:** Microbiome, Colorectal cancer

## Abstract

The primary treatment for non-metastatic colorectal cancer is surgical resection. Despite the use of neoadjuvant and/or adjuvant chemoradiation, up to 30% of patients undergoing surgery for colorectal cancer will develop a postoperative recurrence. Why patients develop postoperative tumors despite all known cancer being resected at the time of surgery is largely unknown, and novel biomarkers that can predict the development of recurrence are lacking. Here, we report a unique bacterial signature present in the gut during the perioperative period that is strongly associated with the development of postoperative tumors. By studying patients undergoing resection for colorectal cancer, we demonstrate that the gut microbiome on the day of surgery is enriched with collagenase-producing bacteria in patients who later develop a recurrence. This bacterial community demonstrated enhanced antimicrobial resistance, was not eradicated by the standardized perioperative bowel preparation, and could promote cancer cell migration and invasion. Our study establishes that microbiota may contribute to postoperative colorectal cancer recurrence and serve as a prognostic biomarker for postoperative oncologic outcomes.

## Abstract (web summary)

Up to one-third of patients develop a cancer recurrence following surgery for colorectal cancer. Here, the authors show that a virulent gut microbial signature present on the day of surgery associates with the development of recurrence in human patients.

## Introduction

With ~153,000 patients diagnosed in 2024^1^, colorectal cancer (CRC) is the third most common cancer diagnosed in the United States. In the current treatment paradigm, once diagnosed with CRC, surgical resection of the primary tumor is almost always required to obtain a long-term cure. Because of how prevalent CRC is, this treatment approach results in over 150,000 patients undergoing CRC surgery annually^2^. However, ~30% of patients undergoing surgical resection will develop a postoperative cancer recurrence either locally at the site of the surgical resection or distally in the liver, lung, or peritoneum^3^. Outcomes in patients developing a recurrence are dismal, with the majority of patients dying within 5 years^4^.

The mechanisms that underscore why certain patients develop postoperative tumors after all known malignant tissue has been resected are poorly understood. We have previously published both clinical and preclinical data identifying the role of bacteria, particularly those that express the enzyme collagenase, in driving CRC recurrence. First, in a single- institution study of 528 patients undergoing curative surgery for CRC at the University of Chicago Comprehensive Cancer Center, a prescription of a combined mechanical bowel preparation to decontaminate the colon perioperatively was independently associated with improved recurrence-free survival compared with patients who did not undergo decontamination^5^. Second, in mice that underwent surgery that recapitulates human CRC resection, enrichment of the perioperative gut microbiome with bacteria that overproduce the enzyme collagenase is highly associated with postoperative tumor formation that mimics human CRC recurrence^6^. Bacterial collagenases are metalloproteinases that are overexpressed under adverse conditions, and therefore, their presence may represent a dysbiotic microbial environment. Whether these collagenolytic microbes, potentially resistant to the effects of a standardized bowel preparation, represent a biomarker and driver of postoperative CRC tumor formation in human patients is unstudied. In this preliminary report, we show that perioperative gut-microbiomes enriched with antimicrobial-resistant collagenolytic bacteria are highly associated with the development of recurrence following CRC surgery.

## Results

### Patients who develop postoperative recurrence have a distinct perioperative microbiome signature

**Fig. 1:**
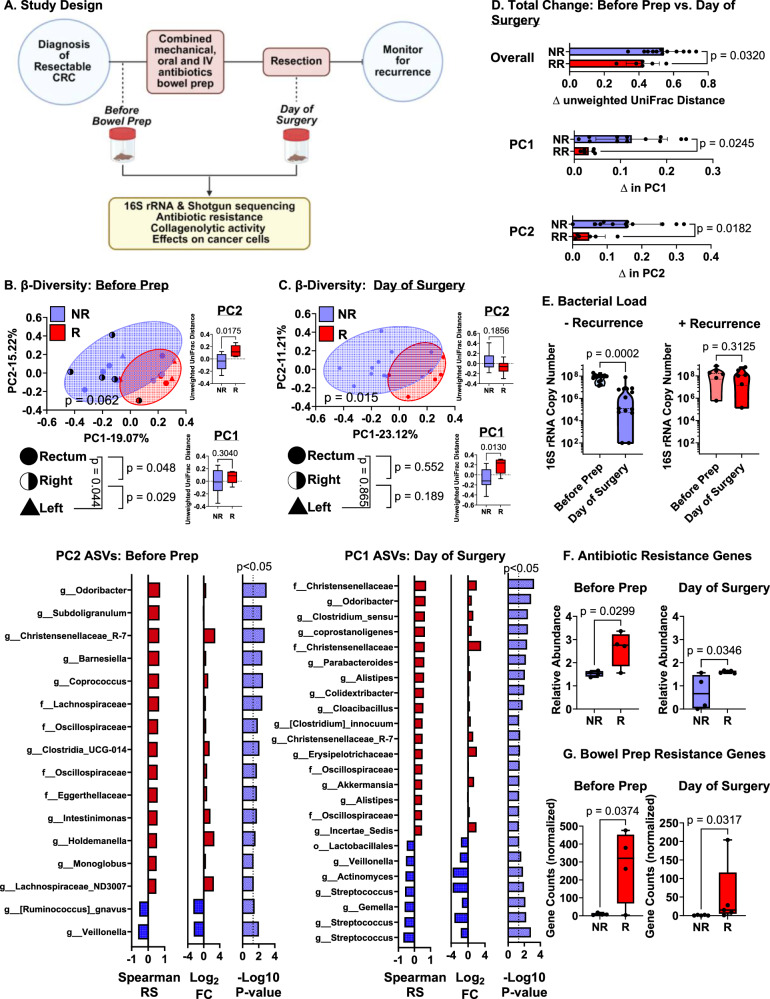
Patients who develop postoperative recurrence have a distinct perioperative microbiome. **A** Study design. Schematic illustration was created in BioRender.com (Created with BioRender. Shogan, B. (2026) https://BioRender.com/t4krvei agreement number JW29JRWW4R). **B** PCoA analysis of unweighted Unifrac distances of stool collected before bowel preparation (top) and the ASVs that are associated with PC2 (bottom). **C** PCoA analysis of unweighted Unifrac distances of stool collected on the day of surgery (top) and the ASVs that are associated with PC1 (bottom). **D** Longitudinal change in Unifrac distances between an individual’s stool recovered prior to bowel preparation compared to the day of surgery. Data are presented as individual data points with median and interquartile range (25th–75th percentiles). *n* = 12 (NR) and *n* = 6 (R) independent samples. **E** Bacterial load of the recovered stool samples. Shotgun metagenomics on stool recovered prior to bowel preparation and on the day of surgery, showing the relative abundance of (**F**) overall antibiotic resistance genes and **G** bowel-prep-specific antibiotic resistance genes (cefoxitin, neomycin, and metronidazole). All boxplots in (**B**, **C**, **F**, **G**) show median (center line), interquartile range (25th–75th percentiles), and whiskers indicating the minimum and maximum values. NR = patients without development of postoperative recurrence. R = patients who develop a postoperative recurrence. *P* values were determined by an unpaired two-tailed t-test (**B**, **C**, **F**, **G**), unpaired two-tailed Mann–Whitney-test (**D**), or two-tailed Wilcoxon test (**E**). Each dot represents one sample size, *n* = 1.

To address this question, we conducted a small prospective study of 19 consecutive patients undergoing surgery for CRC between 3/1/19 and 2/1/20 at the University of Chicago Comprehensive Cancer Center (Fig. 1A). All patients completed a combined preoperative bowel preparation consisting of oral antibiotics (neomycin, metronidazole) and a purgative (MiraLAX) on the day prior to surgery and received intravenous cefoxitin within 30 min of incision on the day of surgery. An expelled stool sample was collected prior to bowel preparation, and a second stool sample was collected on the day of surgery from the opened resected specimen. The patients’ mean age at the time of diagnosis was 64.9 years, 58% were male, 68% had colon cancer, and 32% had rectal cancer. A majority of patients had a tumor grade of 2 (moderately differentiated; 78.9%), and a mean of 16.9 lymph nodes (SD 3.0) were recovered from each surgical specimen. A plurality of patients were clinically staged as stage III (42.1%), followed by stage II (36.8%), and stage I (21.1%). Of the entire cohort, 63.2% of patients received either neoadjuvant or adjuvant therapy. After a median follow-up of 2.0 years (IQR 4.3 years) for the entire cohort, 6 patients (31.6%) developed a postoperative recurrence. The median time from surgery until the diagnosis of recurrence was 10.3 months (IQR 5.0 months). There were no significant differences in demographics, tumor grade, number of lymph nodes resected, stage, or receipt of neoadjuvant/adjuvant therapy between patients who developed a recurrence versus those who did not (Supplementary Table 1). Genetic testing showed that all tumors were microsatellite stable. The most common site of recurrence was the lung (*n* = 3), followed by the liver (*n* = 2) and at the anastomosis (*n* = 1). Two patients developed multisite recurrences (liver/peritoneal and lung/anastomosis). The median length of follow-up for the 13 patients who did not develop a recurrence was 5.1 years (IQR 3.2 years).

Using samples collected from this clinical cohort, we first asked whether differences in the composition of the gut microbiota were unique in patients who would later develop a postoperative recurrence. Specifically, to determine if a microbial signature was present during the perioperative period that would characterize patients at risk for the development of recurrence, we performed 16S rRNA amplicon sequencing on the stool samples recovered prior to bowel preparation and on the day of surgery.

We first analyzed stool samples collected prior to receiving bowel preparation. Not surprisingly, principal coordinate analysis (PCoA) of the unweighted UniFrac distances stratified by tumor location revealed significant differences between the left colon, right colon, and rectal regions (Fig. 1B middle panel). While unweighted UniFrac-based PCoA revealed that there was no significant global difference in the composition of the microbiomes between recurrent and non-recurrent patients (PERMANOVA, *p* = 0.062), significant differences were observed in the PC2 distances (*p* = 0.0175), suggesting that a subset of taxa were distinct between patients (Fig. 1B top panel). To identify the specific microbiota driving these differences, we performed a correlation analysis between the PC2 distances and the rarefied amplicon sequence variant (ASV) read counts and identified 16 ASVs associated with PC2 that potentially drove the differences between patients who developed recurrence and those who did not (Fig. 1B bottom panel). Fifteen ASVs from the genera *Odoribacter, Subdoligranium, Christensenellaceae R-7* group, *Barnesiella, Clostridia UGC-014, Intestinimonas, Holdemanella, and Monoglobus* and families *Lachnospiraceae, Oscillospiraceae, and Eggerthellaceae* were positively associated with the development of recurrence, while two genera, *Veillonella* and *Ruminococcus gnavus,* were associated with non-recurrent patients.

We next asked whether this signature was present on the day of surgery after patients received the prescribed antibiotic bowel preparation. Strikingly, PCoA of the unweighted UniFrac distances of the stool samples collected on the day of surgery revealed a global microbial community composition difference between patients who would later develop a recurrence vs. those that did not (PERMANOVA, *p* = 0.015; Fig. 1C top panel). Because this was primarily driven by PC1 (Mann–Whitney, *p* = 0.013), we performed a correlation analysis between the PC1 distances and the rarefied ASV read counts. Twenty-four ASVs were significantly associated with PC1, of which the same ASV from the genus *Veillonella* was associated with non-recurrent patients, whereas ASVs from the genus *Odoribacter* and the family *Oscillospiraceae* were associated with recurrent patients (Fig. 1C bottom panel). Interestingly, the well-appreciated differences in composition between the left colon, right colon, and rectal regions prior to bowel preparation were no longer observed on the day of surgery (Fig. 1C middle panel). There was no difference in α-diversity (i.e., Shannon’s diversity or evenness) between recurrence vs. non-recurrent patients in either stool samples recovered prior to or on the day of surgery (Supplementary Fig. 1A, B).

Collectively, these findings suggested the presence of a microbial signature that was present both before bowel preparation and after the bowel preparation on the day of surgery in patients who later developed recurrence. As mentioned above, clinical data reveal that patients who undergo preoperative bowel preparation have a decreased incidence of postoperative recurrence^5^. Therefore, to determine the magnitude of changes in the microbiome induced by the bowel preparation, we longitudinally compared each patient’s pre-bowel preparation sample with their own sample recovered on the day of surgery by calculating the distances in the first and second principal coordinates of the unweighted UniFrac-based PCoA (Supplementary Fig. 1C). We found that bowel preparation induced significantly more changes in the overall community structure in non-recurrence patients compared with patients who developed a postoperative recurrence (Mann–Whitney, *p* = 0.032; Fig. 1D top panel). Similarly, bowel preparation induced significantly more changes in both PC1 (Mann–Whitney, *p* = 0.0245; Fig. 1D middle panel) and PC2 (Fig. 1G, Mann–Whitney, *p* = 0.0182; Fig. 1D bottom panel) in patients who did not develop recurrence compared with those who did. In addition to the effect of bowel preparation on bacterial composition, we analyzed its influence on the quantity of bacteria present in the stool samples using qPCR normalized to stool mass. We found that in patients who later developed a recurrence, the total number of bacteria was 23-times greater in the stool collected on the day of surgery compared with those who did not develop a recurrence (16S rRNA copy number ± SD: 1.9 × 10^8^ ± 2.1 × 10^8^ vs. 8.1 × 10^6^ ± 2.2 × 10^7^; Mann–Whitney, *p* = 0.0032). By longitudinally comparing each patient’s pre-and post-bowel preparation samples, we found that in non-recurrence patients, bowel preparation resulted in a 10-fold decrease in the total bacterial concentration (16S rRNA copy number ± SD: 8.3 × 10^7^ ± 5.1 × 10^7^ vs. 8.1 × 10^6^ ± 2.2 × 10^7^; Wilcoxon signed-rank test, *p* = 0.002; Fig. 1E left panel), whereas bowel preparation had no significant effect on bacterial load in recurrence patients (16S rRNA copy number ± SD: 2.5 × 10^8^ ± 2.6 × 10^8^ vs. 1.9 × 10^8^ ± 2.1 × 10^8^; Wilcoxon signed-rank test, *w* = 5, nonsignificant; Fig. 1E right panel).

### The microbiomes of patients who develop postoperative recurrence have an enrichment of antibiotic resistance genes

Given that patients who developed recurrence appeared to be resistant to the effects of the bowel prepation, we next tested the stool samples for antibiotic resistance by performing shotgun metagenomics for the presence of bacterial antibiotic-resistance genes. Strikingly, we found that both the total number of antibiotic-resistance genes (Fig. 1F) and the number of resistance genes specific to the three antibiotics given as part of the bowel preparation (cefoxitin, neomycin, and metronidazole) were significantly greater in patients who developed recurrence compared to those that did not, both prior to bowel preparation and on the day of surgery (Fig. 1G). To determine if the presence of these genes conferred functional resistance to the bacterial community in vitro, we cultivated each patient sample and tested for susceptibility to cefoxitin and neomycin on agar plates and in liquid broth. We found that the mean susceptibility of the day of surgery samples to cefoxitin was significantly higher in patients who developed a postoperative recurrence compared with those who did not (Mann–Whitney, agar plate *p* = 0.0105, liquid broth *p* = 0.0216; Supplementary Fig. 2A right panel). However, despite the enrichment of antibiotic resistance genes in these samples, we did not observe functional resistance to either cefoxitin prior to bowel preparation (Supplementary Fig. 2A left panel) or to neomycin in either pre-bowel preparation or day-of-surgery samples (Supplementary Fig. 2B). There are multiple explanations for this discrepancy. First, functional deviation from expected metagenomic antibiotic resistance determinants is common and could be due to changes in in vitro gene expression^7^. These changes may be driven by the loss of key host components, environmental conditions, community interactions, spatial structure, and biofilm physiology that exist in situ^8–10^. Second, there are likely changes in community composition and species richness following outgrowth in laboratory media, making the substrate for the functional assays potentially different from the substrate for the genomic assays. Finally, the fact that we observed functional resistance to cefoxitin, but not to neomycin, could be explained by the phenomenon whereby cefoxitin resistance mechanisms protect susceptible species by detoxifying the antibiotic and therefore have a significant impact on cefoxitin resistance within the community^11^. This has been described in relation to AmpC-mediated β-lactam resistance, but has not been observed for aminoglycoside-modifying enzymes that similarly modify aminoglycoside antibiotics such as neomycin^12^. Yet despite these limitations, the antibiotic resistance analyses, particularly the metagenomic analysis, reveal the presence of an antibiotic-resistant bacterial community associated with the development of recurrence.

### The microbiome on the day of surgery in patients who develop a postoperative recurrence is enriched with collagenase-producing bacteria

**Fig. 2:**
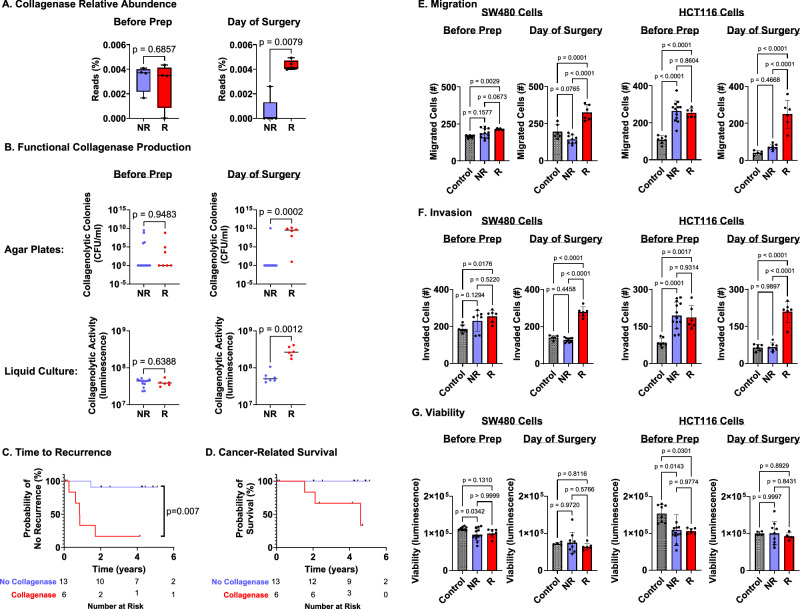
The gut microbiome on the day of surgery in patients who develop a postoperative recurrence is enriched with collagenase-producing bacteria that promote tumor progression of cancer cells. **A** Shotgun metagenomics for bacterial collagenase genes on stool recovered prior to bowel preparation and on the day of surgery. The center line shows the median, the box shows the interquartile range (25th–75th percentiles), and the whiskers indicate the minimum and maximum values. **B** Functional collagenase activity of in vitro cultivated stool samples was measured on agar plates overlaid with milk (upper panel) and by coincubation with collagen in liquid culture (lower panel). Time-to-event analysis between the presence of functional bacterial collagenase and the development of recurrence (**C**) and cancer-related survival (**D**). The effect of bacterial cell-free supernatants on cancer cell migration (**E**), invasion (**F**), and proliferation (**G**) of human-derived SW480 and HCT116 cancer cells. For **E**–**G**, the line indicates the mean and error bars indicate  ± standard deviation. NR = patients without development of postoperative recurrence. R = patients who develop a postoperative recurrence. *P* values were determined by an unpaired two-tailed Mann–Whitney-test (**A**, **B**) or one-way ANOVA with a multiple comparison test (**E**–**G**). For **B** (agar), each dot represents the mean of two biological replicates from a single patient. For **B** (liquid), each dot represents the mean for three technical replicates from a single patient. For **E** and **F**, each dot represents the mean of five representative fields from a single patient. For **G**, each dot represents the mean of four technical replicates from a single patient.

As we have previously identified enrichment of bacterial collagenases as being associated with post-surgical recurrence, we next assessed the collagenolytic potential of the microbiomes^6^. Shotgun metagenomics of stool collected prior to bowel preparation showed that there was no difference in either the relative abundance of genes (Mann–Whitney, *p* = 0.6857; Fig. 2A) or the number of genes responsible for collagenase production between patients who developed recurrence and those who did not (Mann–Whitney, number of genes: *p* = 0.4857; Mann–Whitney, read depth: *p* = 0.3429; Supplementary Fig. 3A). Conversely, analysis of stool collected on the day of surgery following the attempted bowel preparation demonstrated that the microbiome had both a significantly increased relative abundance (Mann–Whitney, *p* = 0.0079; Fig. 2A) and number of collagenase-producing genes (Mann–Whitney, number of genes: *p* = 0.0317; Mann–Whitney, read depth: *p* = 0.0159; Supplementary Fig. 3A). Given these genomic differences, we tested the bacterial communities for their functional collagenolytic activity in vitro via two complementary methods that we have previously reported^13^: (1) plating bacterial samples on agar overlaid with skim milk in which colonies with a clear halo are inferred to possess collagenolytic activity and (2) by directly co-incubating bacterial samples with fluorescent gelatin, the denatured form of collagen, and measuring its breakdown in liquid broth. Corroborating the metagenomic results, bacterial collagenolytic activity on the day of surgery was significantly higher in patients who later developed recurrence compared with patients who did not in both agar and liquid culture assays (Fig. 2B right panel). In fact, there were no detectable collagenolytic colonies in 12 of the 13 patients who never developed a recurrence. In samples collected prior to surgery, there was no difference in collagenolytic activity between recurrence and non-recurrence patients, suggesting that the antibiotic bowel preparation was required to uncover this virulent phenotype (Fig. 2B left panel).

Given the dramatic difference in collagenolytic activity on the day of surgery, we analyzed the association of functional collagenase activity on the day of surgery with oncological outcomes. Time-to-event analysis demonstrated that the presence of functional bacterial collagenase on the day of surgery was significantly associated with the development of a postoperative recurrence (HR 17.0; 95% CI 1.9–149.0; log-rank *p* = 0.007; Fig. 2C). We next wanted to adjust for potential confounders that would influence recurrence. Due to the small number of recurrence events, we could not adjust for covariates in the Cox regression model and therefore performed an exact logistic regression for the dichotomized outcome of the development of recurrence or its absence, adjusting for potential confounders influencing recurrence outcome^14^. Using this model, we found a similarly significant association of collagenase activity with the development of recurrence (OR 38.6; *p* = 0.006). Five subsequent independent analyses were conducted, adjusting for receipt of neoadjuvant/adjuvant therapy, clinical AJCC stage, pathological AJCC stage, tumor grade, and number of lymph nodes recovered. Our results show that a significant association between collagenase activity and recurrence remained when adjusting for the receipt of neoadjuvant/adjuvant chemotherapy or radiation (adjusted OR 20.2; *p* = 0.005), pre-neoadjuvant therapy clinical AJCC stage (adjusted OR 29.4; *p* = 0.003), post-resection pathological AJCC stage (adjusted OR 18.2; *p* = 0.019), tumor grade (adjusted OR 26.0; *p* = 0.015) and number of lymph nodes removed (adjusted OR 14.9; *p* = 0.008). Similarly, cancer-associated mortality was higher in patients who harbored collagenase activity compared to patients who did not (Fig. 2D). Taken together, this analysis of antibiotic sensitivity and collagenase activity suggests that the perioperative bowel preparation uncovered an antibiotic-resistant collagenolytic microbiome that is significantly and independently associated with the later development of recurrence.

### Supernatants from collagenolytic microbiomes recovered from patients who develop postoperative recurrence promote tumor progression in cancer cells

We have previously shown that *Enterococcus faecalis*, a common collagenase-producing gut commensal, can promote cancer cell invasion via collagenase-dependent pathways and promote cancer cell migration via collagenase-independent pathways in vitro^15^. Therefore, we assessed how secreted products of these human-derived bacterial communities could promote tumor progression by co-incubating them with cancer cells. We found that supernatant from those collagenolytic bacterial communities recovered on the day of surgery from recurrence patients significantly increased both migration (Fig. 2E and Supplementary Fig. 4A) and invasion (Fig. 2F and Supplementary Fig. 4B) of both human-derived cancer cells (SW480, HCT116) and mouse-derived cancer cells (CT26, MC38) compared with the non-collagenolytic bacteria collected from non-recurrence patients. There was no significant difference in cancer cell migration and invasion between recurrence and non-recurrence patients when the pre-bowel preparation samples were used, similarly indicating that in patients who later developed a recurrence, the perioperative bowel preparation uncovered the presence of a collagenolytic microbiome that could promote cancer cell migration and invasion. Next, we assessed whether the effect of bacterial communities on cancer cell migration and invasion was due to enhanced proliferation of cancer cells or rather to an intrinsic shift of cancer cells to this more metastatic phenotype. Proliferation assays revealed no effect of the bacterial communities on cancer cell growth, implicating a bacterial-induced switch of cancer cells towards a migratory and invasive phenotype (Fig. 2G).

### Collagenase-producing strains isolated from patients who develop postoperative recurrence promote cancer cell migration and invasion

We next sought to identify specific collagenolytic bacterial strains within the bacterial community that might be responsible for promoting enhanced migration and invasion. To do this, we speciated the stool samples collected on the day of surgery from all of the patients who developed recurrence on non-selective agar plates overlaid with skim milk. We then picked colonies with a halo (indicating collagenase activity) and those without a halo (indicating no collagenase activity) from each patient (10–12 colonies per patient, for a total of *n* = 72 colonies) to make a bacterial strain library. Collagenase activity was then confirmed by growing each isolate in liquid broth, and Sanger sequencing was used to identify the identity of each isolate. Similar to our previous work^6^, *Enterococcus* genus was the predominant genus in this 72-strain library (*n* = 33; 45.8%), and we identified both collagenase-producing and non-collagenase strains of *E. faecium*, *E. durans*, and *E. avium* (Supplementary Table 2). To determine whether these individual strains could promote cancer progression, we selected collagenase-producing and non-collagenase-producing strains from each of these species and performed in vitro migration and invasion assays. In addition, because it exhibited the highest collagenase activity among the strains, we tested a strain of *Pseudomonas aeruginosa*. Similar to when the entire bacterial community was assayed above, we found that the supernatant from those collagenolytic bacterial strains significantly increased both migration (Supplementary Fig. 5A) and invasion (Supplementary Fig. 5B) of human-derived cancer cells (SW480, HCT116), whereas non-collagenolytic strains had no effect. Proliferation assays showed no effect of the individual strain on cancer cell growth, suggesting a bacterial-induced switch in cancer cells toward a migratory and invasive phenotype (Supplementary Fig. 5C).

### Genomic differences exist between the collagenase-producing and non-collagenase-producing strains

Finally, whole genome sequencing was performed on these seven isolates, and their assembled sequences were annotated. This confirmed both the taxonomic identity described by the Sanger sequencing and the purity of a single strain that was used in the in vitro assays (Supplementary Fig. 6). To inspect the genomic differences between the collagenase-producing and non-collagenase-producing strains, we performed a strain-to-strain pangenomic analysis for each species with its matched pair. Comparisons revealed that each species ranged in their degree of overlap of their core genomes (*E. faecium* 93.96% average nucleotide identity (ANI), *E. durans* 98.37% ANI, *E. avium* 99.98% ANI; Supplementary Fig. 7). Furthermore, there were 8961 variant alleles between the *E. faecium* collagenase-producing and non-collagenase-producing strains, 1865 variant alleles between the *E. durans* collagenase-producing and non-collagenase-producing strains, and 44 variant alleles between the *E. avium* collagenase-producing and non-collagenase-producing strains; these variant alleles were of significant divergence to be considered noncore genes. Functional annotation revealed that all collagenase-producing strains demonstrated a more diverse genome with a larger metabolic capacity, and increased species-specific genes involved in virulence, such as the type IV secretion system (PF16943), adhesion proteins (PF05738), and staphopain peptidase C47, which breaks down collagen (PF05543) (see Supplementary File 1 for a complete list of gene differences). We found no differences in any other genes involved in the regulation and production of collagenase-producing and non-collagenase-producing strains.

## Discussion

Taken together, while validation in a larger cohort is needed, this preliminary report provides evidence that enrichment of the perioperative gut-microbiome with collagenolytic bacteria that can promote a migratory and invasive cancer cell phenotype is highly associated with the development of postoperative tumors. This bacterial signature was detected on the day of surgery, only after the patient received attempted antibiotic decontamination. This indicates that while these bacteria must have been present prior to decontamination, removal of the other commensal bacteria was required to detect these virulent microbes within the laboratory. While we did not directly assess for tumor-associated microbiota, we have previously shown that collagenolytic organisms preferentially bind to tumor tissue^15^. Because the day-of-surgery, the sample was collected from residual stool that was in contact with the tumor, we speculate that there was an interaction of this microbiota with the growing tumor preoperatively. Given our data showing that this collagenolytic community can promote tumor progression in vitro, it is plausible that this tumor–microbe interaction promotes an aggressive tumor phenotype or drives micrometastatic disease prior to surgery, which is then identified as a recurrence postoperatively. Follow-up studies directly investigating the bacteria colonizing the tumor will be needed.

How bacteria with a collagenolytic phenotype, interacting with growing tumor tissue, promote cancer progression remains unclear, but both direct and indirect mechanisms are likely involved. Here, we identified that strains of *E. faecium*, *E. durans*, and *E. avium* promoted cancer cell migration and invasion in vitro in a collagenase-dependent manner. While *Enterococcus* spp. has been implicated in the pathogenesis of CRC, the dependence of its tumorigenesis on a collagenolytic phenotype has not been addressed^16^. We have previously shown that collagenolytic *E. faecalis* changes the transcriptional profile of cancer cells, particularly integrin-mediated signaling, leading to cancer progression^15^. Additionally, collagenolytic organisms can bind to and activate components of the uPA-plasminogen system, a well-known pathway known to be associated with CRC metastasis and postoperative tumor formation^17–21^. Further, collagenase can affect angiogenesis and immune response by remodeling the tumor cell extracellular matrix^22^. Whole genome sequencing of the collagenolytic *Enterococcus* strains did not demonstrate any genomic differences in genes known to regulate collagenase production. This lack of genomic difference in collagenase machinery is consistent with our previous comparison of high and low collagenase-producing *E. faecalis* strains recovered from rats^23^. However, collagenase-producing strains that promoted in vitro cancer progression in the current study had larger genomes with greater genomic potential for virulence and pathogenesis compared to matched strains that were non-collagenase-producing in vitro. Follow-up studies are needed to elucidate the mechanisms by which these genetic differences lead to differential collagenase expression.

Given the effect of collagenolytic bacteria on promoting a migratory and invasive cancer cell phenotype, we have targeted them with an antibiotic-directed approach as a strategy to decrease postoperative tumors^6^. Although we have shown that certain collagenolytic strains can be eradicated, other collagenolytic species emerge, suggesting the presence of a unique ecological niche for collagenase-producing bacteria that arises under certain conditions. What drives the creation of this environment and developing more ecologically favorable ways to prevent the emergence of this phenotype are under investigation.

While the aforementioned in vitro analysis demonstrates that the collagenolytic microbiota, identified on the day of surgery in patients who later develop recurrence, promotes tumor progression, it is important to acknowledge the limitations of these experiments. The outgrowth of patient samples in laboratory medium is likely to result in an altered microbial composition and species richness compared with the microbial community in stool samples. These cultivated samples were used for the in vitro assays due to the variability in the amount of stool donated by the subject and the concern that an adequate amount of the original sample would not be available to complete all the in vitro experiments. However, the fact that there is consistency between our metagenomic analyses and in vitro results for both the presence of collagenase genes and functional collagenolytic activity suggests that these assays recapitulate an important, biologically relevant characteristic of the microbial population, which is consistent with our previous work. Furthermore, the identification of individual collagenase-producing bacterial strains within the collagenolytic communities that are associated with recurrence gives further evidence that this bacterial phenotype may play a key role in CRC recurrence pathogenesis.

In conclusion, our preliminary report supports a potential link between bowel prep-resistant collagenolytic microbiota and the development of postoperative CRC recurrence in human patients. Furthermore, this study highlights the potential of the gut microbiota as both a diagnostic tool for identifying critically vulnerable patients and a modifiable therapeutic target to improve CRC outcomes. However, while our findings may have future clinical relevance, given our limited sample size and potential confounding, our findings need confirmation in a large, multi-institutional study. Nevertheless, as a first report, our study lays the foundation for understanding how a collagenolytic bacterial signature is both a driver and a marker of poor oncological outcomes following surgery for CRC.

## Methods

This study was conducted in accordance with all relevant ethical regulations. The study protocol was reviewed and approved by the University of Chicago Institutional Review Board (IRB 17-0417).

Patient population: all subjects provided informed consent and did not receive compensation. Patients aged ≥18 years old undergoing an elective colorectal resection for malignancy at the University of Chicago Comprehensive Cancer Center were recruited to participate in this prospective study. All recruited patients underwent a preoperative bowel preparation with a mechanical purgative (polyethylene glycol) combined with oral neomycin (1000 mg, 3 doses) and oral metronidazole (500 mg, 3 doses) on the day prior to surgery. On the day of surgery, each patient received intravenous cefoxitin (2 gm) 30 min prior to surgical incision. Patients were excluded if they currently had an ostomy or had a contraindication to receiving a combined bowel preparation. Sex and race for each subject were self-reported from predetermined lists: male/female/other; Caucasian/African American/Asian/American Indian/Alaska Native/Native Hawaiian/Pacific Islander/Middle Eastern.

Development of postoperative recurrence: Patients were followed for 5 years postoperatively at the University of Chicago Comprehensive Cancer Center for the development of recurrence using the guidelines published by the National Comprehensive Cancer Network. The electronic medical record was reviewed for each patient, and the development of recurrence was determined if the patient’s oncologist documented recurrence.

Stool sample collection: two stool samples were collected from each patient. The first sample was collected preoperatively, prior to the administration of the mechanical and antibiotic bowel prep, and is referred to as the “*before bowel prep*” sample. The second sample was acquired from the resected specimen in the pathology lab on the day of surgery, and is referred to as the “*day of surgery*” sample. The “before bowel prep” samples were collected using the BioCollector stool kit (BioCollective). The “day of surgery” sample was collected from the surgically resected specimen on the day of surgery. To do this, once the specimen was physically resected from the patient, the clinical research coordinator immediately carried it from the operating room to the University of Chicago Pathology Department Human Tissue Resource Center (approximately 5-min walk). Here, a dedicated Human Tissue Resource Center technician, trained in this specific protocol, performed the following standardized procedure to obtain intraluminal stool: first, the specimen was opened longitudinally, away from the tumor, to avoid contamination. Once the specimen was opened, the residual stool on top of and in contact with the tumor was spooned into a sterile cup using a sterile spatula. Stool that was not in contact with the tumor or on the margins was not collected, and to avoid cross-contamination, the lumen was not flushed or irrigated prior to stool collection. All samples were processed immediately by homogenizing them in lysing matrix D (MP Biomedicals, 6913100). Aliquots of homogenized stool were stored either alone or mixed with 20% glycerol at −80 °C until they were used for future experiments.

Individual bacterial species/strain identification: to identify bacterial strains within the stool bacterial community that could promote cancer progression, we first cultured stool collected on the day of surgery from all of the patients who developed recurrence on TSB plates overlaid with skim milk (see below “*Collagenolytic activity*” for details). We then randomly picked approximately 10–12 colonies from each patient (a total of 72 colonies picked), some of which had a halo indicating collagenolytic activity, whereas others did not have a halo, indicating no collagenolytic activity. A small amount of colony from the plate was resuspended in 20 μl of TSB medium and after 1:10 dilution, 2 μl of this broth was used to amplify the 16S rRNA region using a PCR master mix kit (Qiagen, cat # 201443) and bacteria specific-16S rRNA primers (forward primer, 5′ 27F: 5′- AGA GTT TGA TYM TGG CTC AG -3′, reverse primer, 1492R: 5′- TAC GGY TAC CTT GTT ACG ACT T -3′) with the following PCR conditions; initial denaturation/lysis at 95 °C for 10 min, denaturation at 94 °C for 30 s, annealing at 56 °C for 30 s, and extension at 72 °C for 1.5 min with total 40 cycles followed by final extension at 72 °C for 10 min. Each PCR product was confirmed on agarose gel as a single band (~1.5 kb), and after using the PCR purification kit (Qiagen, cat #28106), samples underwent Sanger Sequencing at the University of Chicago Genomics core using bacteria-specific 16S rRNA primers (forward primer, 341F: 5′- CCT ACG GGN GGC WGC AG-3′ and reverse primer, 1378R: 5′- CGG TGT GTA CAA GGC CCG GGA ACG-3′). Resulting sequences were blast-matched for identification. Glycerol stocks for each identified colonies were made by growing the picked colony in TSB broth overnight at 37 °C. This glycerol stock was used for subsequent collagenase, migration, invasion, and viability assays as described below.

16S rRNA sequencing: 16S rRNA amplicon sequencing was performed in the University of Chicago Duchossois Family Institute Microbial Metagenomic facility (DFI-MMF). DNA from homogenized stool (non-glycerol aliquots) was extracted using the QIAamp PowerFecal Pro DNA Kit, and a V4–V5 region of the 16S rRNA gene was PCR amplified with primers (Fwd-AYTGGGYDTAAAGNG) and (Rev-CCGTCAATTYHTTTRAGT). 16S rRNA copy number normalized to stool mass was used to determine bacterial load. Illumina-compatible libraries were generated using the Qiagen QIASeq 1-step amplicon kit and sequenced on the Illumina MiSeq platform, generating 2 × 250 PE reads and >10,000 reads per sample, and analyzed on the Qiime2 platform (v2024.5)^24^. Briefly, FASTQs were demultiplexed and quality filtered via the DADA2 algorithm with truncation of all reads to 190 bp to generate ASV counts and representative sequences. Taxonomic classification was performed using a Bayesian RDP classifier trained on 16S sequences from the SILVA database (v138_2)^25^. Alpha and beta diversity metrics and PCoA plots were generated using the command “*qiime diversity core-metrics.”* Statistical significance between non-recurrence (NR) and recurrence (R) was calculated with commands “*qiime diversity alpha-group-significance”* and “*qiime diversity beta-group-significance”* for alpha diversity (Mann–Whitney) and beta diversity (PERMANOVA), respectively^18^. To determine ASVs associated with specific principal components (PCs), Spearman rank coefficients were calculated between the unweighted UniFrac distance-based sample PC coordinates and the rarefied ASV counts +1, filtering for associations *p* < 0.05. Change in the microbiome due to bowel prep was quantified for each patient as the unweighted UniFrac distance or PC distance between the day of surgery sample and the sample before bowel prep.

Shotgun metagenomics: shotgun metagenomics was performed at the DFI-MMF. Libraries for shotgun metagenomics were prepared using 100 ng of genomic DNA using the QIAseq FX DNA library kit (Qiagen). Fragmented DNA was end-repaired with 3′ poly-A addition as staging inserts for ligation. Illumina unique dual index adapters were ligated to the inserts, and PCR amplified and bead purified with QC performed on Tapestation (Agilent Technologies). Libraries were sequenced on an Illumina NextSeq 500 to generate 2 × 150 reads. Adapters were trimmed off the raw reads with low quality bases (PHRED < 30) with Trimmomatic v.0.39^26^. Reads mapping to the human genome were removed by kneaddata v0.7.10 (https://github.com/biobakery/kneaddata). Taxonomic profiling was performed using Metaphlan4^27^. Single-sample assemblies were generated using MEGAHIT v1.2.9, with resulting contigs imported and analyzed in the Anvi’o platform (v8). Contigs <2000 bp were removed from each sample before short reads were mapped to the contigs (bowtie2, samtools). Open reading frames were identified using Prodigal (v2.6.3) by executing the anvio command “anvi-gen-contigs-database,” and functionally annotated using the Kyoto Encyclopedia of Genes and Genomes (KEGG) for orthologous genes (“anvi-run-kegg-kofams”) and protein families database (PFAM, “anvi-run-pfams”). Q2Q3 gene coverages were calculated using “anvi-export-gene-coverage-and-detection”.

Whole genome sequencing of individual bacterial isolates: whole genome sequences was performed on an Illumina MiSeq. Short reads were assembled into contigs (*MEGAHIT),* and shorts reads were mapped to the contig reference (*bowtie2)*. Contigs and reads were then analyzed on the Anvio’o platform (v8)^28^, with taxonomic (*kaiju)* annotations made to confirm congruent assignment of all the contigs. Each genome contained <5% redundancy and 100% completion. Variant alleles of significant divergence to be considered a noncore gene was analyzed using the mcl parameter 10 for strain comparisons^29^. Functional annotation was performed using “*anvi-run-kegg-kofams*”and “*anvi-run-pfams*” to generate the KEGG ortholog family and protein family annotations for each gene call. Pangenomics was performed according to Delmont and Eren^30^ using parameters minbit = 0.5 and mcl-inflation = 10 for within species com. Functional enrichment between strains was calculated using the Anvio program “*anvi-compute-functional-enrichment-in-pan*” to identify shared and unique gene clusters.

Bacteria culture and preparation of bacterial cell-free supernatant: bacteria were revived from the glycerol stock aliquots of stool or the individual isolate by culturing in 2 ml of tryptic soy broth (TSB; Remel, R455054) medium at 37 °C for ~12 h. To normalize the number of bacteria used to create the supernatant, cultured bacteria was diluted in fresh TSB to obtain an OD (600 nm) = 0.5. To make cell-free supernatants, the bacterial culture was centrifuged (2500 × *g*, 10 min, 4 °C) to separate bacterial cells and the secreted fraction (supernatant), and the supernatant was then sterilized using 0.2 µm filter. This cell-free supernatant was used for migration, invasion, and cell viability experiments.

Antibiotic resistance: we tested the susceptibility of the antibiotics used in bowel prep on both agar and in liquid culture. First, each glycerol stool sample was grown in 2 ml of TSB for 2 h at 37 °C to allow for recovery. For agar plate testing, 100 µl of this culture was inoculated onto a Mueller-Hinton agar plate. Susceptibility to cefoxitin was determined using MIC test strips (Liofilchem, 92066), and Oxoid™ Neomycin Antimicrobial Susceptibility discs (Thermo Scientific™, CT0033B) were used for neomycin. These antibiotic test strips or disks were placed on each agar plate, and MIC (for cefoxitin) and the zone of inhibition ZOI (for neomycin) were measured after 24 h of incubation at 37 °C. For samples that showed resistance to the highest concentration of the cefoxitin disk (256 mg/ml), agar plates overlayed with higher concentrations of cefoxitin (up to 1024 mg/ml) were used to determine the MIC. For liquid culture testing, 10 µL of the revived bacterial culture was added to 190 µL of TSB in a 96-well microtiter plate with differing concentrations of cefoxitin (range 0–256 µg/mL) or neomycin (range 0–4096 µg/mL). The 96-well plates were incubated at 37 °C for 20 h, and the OD (600 nm) was measured hourly. The MIC was determined by identifying the lowest antibiotic concentration at which there was no bacterial growth. Because the antimicrobial effect of metronidazole is specific to anaerobic bacteria, metronidazole resistance was not assayed. To identify antibiotic resistance genes, amino acid sequences for each gene (anvi-get-sequences-for-gene-calls) were referenced against the Comprehensive Antibiotic Resistance Database^31^ (blastp). Cefoxitin, metronidazole, and neomycin resistance-associated genes were identified by searching annotations (*grep*) with keywords “cefoxitin,” “metronidazole,” “neomycin,” “cephalosporin,” “AmpC,” “mecA,” or “cmy—2”.

Collagenolytic activity: collagenolytic activity was measured in two ways. First, collagenolytic activity was measured using an EnzChek gelatinase/collagenase assay kit (Invitrogen E12055). Bacteria were revived from the glycerol stock aliquots of stool or the individual isolate by culturing in 2 ml of liquid TSB for 2 h at 37 °C. One hundred ninety microliters of the liquid broth was plated in a well of an opaque 96-well plate and coincubated with 10 µl fluorescent gelatin for 18 h at 37 °C. Fluorescence and OD (600 nm) were measured after 15 h of incubation. Wells with TSB medium only were used as negative controls and subtracted from the experimental wells. *Pseudomonas aeruginosa* strain (PA01), a known collagenolytic strain, grown in liquid TSB was used as a positive control. Collagenolytic activity was normalized to cell density via the OD (600 nm) measurement.

Second, we used skim milk plates as previously described^13^. To make these plates, 30 g of the TSB and 15 g agar (BD Difco™ Plate Count Agar, BD 247940) were dissolved in 1 L of distilled water, sterilized by autoclaving at 110 °C for 15 min, and cooled at 45–50 °C. Fifteen percent skim milk (Difco, 232100) was autoclaved for 5 min, and 100 ml of it was added to the cooling agar (1:10 dilution), mixed, then poured into sterile petri plates. To test collagenolytic activity, each glycerol stool sample was first grown in 2 ml of TSB for 2 h at 37 °C. Serial dilutions were then created in TSB, and 100 µl of culture was inoculated onto the milk plate and incubated at 37 °C for 24 h. Colonies that developed a halo around them represent collagenolytic colonies and were counted. Bacterial colony-forming unit (CFU/ml) was calculated.

Cell lines: human-derived cell lines HCT116 and SW480 were kindly provided by Dr. Christopher Weber at the University of Chicago. Mouse-derived colon cell lines CT26 and MC38 were purchased from ATCC (CRL-2638) and Kerafast (ENH204-EP), respectively. Each cell line was maintained as recommended by ATCC: HCT116 cells in McCoy’s 5A media, SW480 cells in Leibovitz’s media, CT26 cells in RPMI media, and MC38 cells in DMEM media. All media was supplemented with 10% fetal bovine serum and 1% penicillin/streptomycin. Additionally, DMEM media for MC38 cells was supplemented with 2 mM glutamine, 0.1 mM nonessential amino acids, 1 mM sodium pyruvate, 10 mM Hepes, and 50 µg/ml gentamycin sulfate. All cells were routinely tested for mycoplasma contamination using the MycoAlert Mycoplasma Detection Kit (Lonza #LT07-118).

Migration and invasion assay: creation of cell-free supernatants (CFS) is described above and was used for migration and invasion assays. For migration assays, 3 × 10^5^ HCT116 cells or SW480 cells or 1 × 10^5^ CT26 or MC38 cells in 150 μl of their respective serum-free media were loaded into the upper chamber of a transwell (Falcon, 353097), and 50 μl of CFS (25% of the total volume) was added to the cells. The lower chamber was filled with 700 μl medium containing 10% FBS as a chemoattractant. After 24 (HCT116 and CT26 cells) or 48 h (SW480 and MC38 cells) of incubation at 37 °C, migrated cells were fixed with 4% paraformaldehyde and stained with 0.5% crystal violet. Invasion assays were performed in an identical fashion except that transwells were coated with 50 μg/cm^2^ of rat tail collagen I (Gibco, A10483-01) for 3 h at 37 °C prior to exposure of the bacteria-cancer cell solution. For each sample, 5 representative fields of view of migrated or invaded cells were pictured using AmScope microscopy at ×20 magnification and counted using the multipoint tool on ImageJ (version 13.0.6). The mean number of cells counted in the 5 fields was calculated and represented as a dot in the figure. Control wells were performed in an identical fashion, except that TSB was placed instead of CFS.

Cell viability: 3 × 10^4^ HCT116 or SW480 cells or 1 × 10^5^ CT26 or MC38 cells cultured in serum-free media were co-incubated with 25% CFS (creation of CFS described above), plated on 96-well opaque plates, and incubated for 24 (HCT116 and CT26 cells) or 48 h (SW480 and MC38 cells) at 37 °C. After incubation, cell viability was measured using the CellTiter-Glow kit (Promega, G9241). Viability assays were performed in parallel with the migration or invasion assays using the identical sets of cells and CFS. Each dot represents the mean of quadruplicate wells. Control wells were performed in an identical fashion, except that TSB was placed instead of CFS.

Statistics and reproducibility: experiments were conducted using three independent biological replicates unless otherwise stated and repeated in at least two independent experiments. Samples were randomized prior to analysis, and investigators were blinded to group allocation during data collection. No statistical method was used to predetermine the sample size, and no data were excluded from the analysis. Statistical analyses were performed using GraphPad Prism software (version 10.5.0) for antibiotics, collagenase activity, invasion, and migration assays. Comparison of unpaired groups was performed using the Mann–Whitney test or Wilson signed-rank for paired groups. Comparison of proportions was performed using Fisher’s exact tests Wilcoxon rank-sum tests were used for bacterial alpha diversity, and permutational multivariate analysis of variance (PERMANOVA) and Kruskal–Wallis tests were used for bacterial beta diversity. An exact logistic regression for the dichotomized outcome of whether a recurrence occurred or did not occur was conducted^14^. *P* < 0.05 were considered statistically significant.

### Reporting summary

Further information on research design is available in the Nature Portfolio Reporting Summary linked to this article.

## Supplementary information

Supplementary Information

Reporting Summary

Transparent Peer Review File

## Source data

Source Data

## Data Availability

All data is publicly available. The 16S rRNA, shotgun metagenomics, and whole genome sequencing data generated in this study have been deposited in the NCBI Sequence Read Archive (SRA) under project number PRJNA1233323. All other data generated in this study have been deposited in Zenodo under 10.5281/zenodo.20633311 (zenodo.org)^32^. All other data supporting this study are available at or are present within the manuscript. Source data are provided with this paper and deposited in Zenodo under 10.5281/zenodo.20633311 (zenodo.org)^32^. Source data are provided with this paper.
