# Supplementary material for: A virulent bacterial signature is associated with the development of recurrence following colorectal cancer surgery
Source: Nat Commun. 2026 Jul 6;17:8361. doi: 10.1038/s41467-026-74889-x (PMC13473480; doi:10.1038/s41467-026-74889-x)
Supplement: Supplementary file 1 [file 41467_2026_74889_MOESM1_ESM.pdf]

Title:

A virulent bacterial signature is associated with the development of recurrence following colorectal cancer surgery

The PDF file includes:

1. Supplementary Figures 1 to 7

2. Supplementary Tables 1 to 3

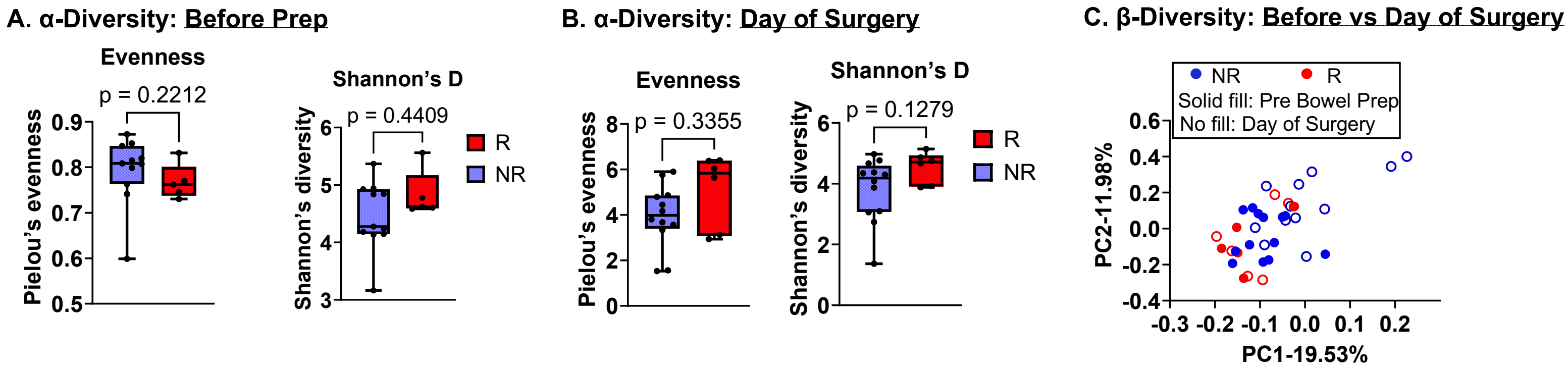

Supplementary Figure 1

A. Alpha diversity of stool collected before bowel prep. **B.** Alpha diversity of stool collected on the day of surgery. **C**. PCoA analysis of both stool collected before bowel prep and on the day of surgery. Each dot represents one sample. NR=patients without development of postoperative recurrence; R=patients who develop a postoperative recurrence. P values were determined by unpaired two-tailed Mann–Whitney-test (A, B). Each dot represents one sample size, n=1.

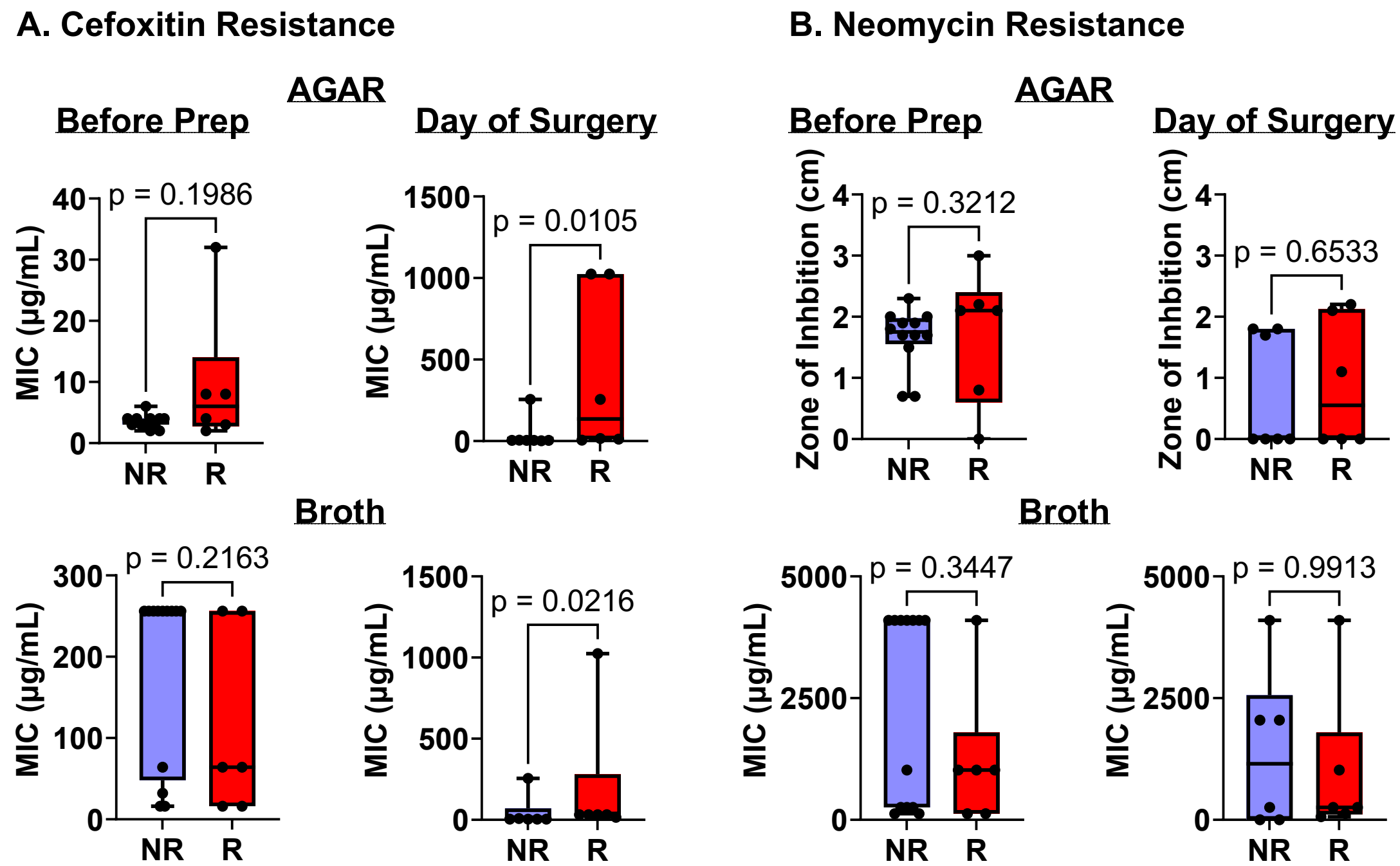

Supplementary Figure 2

A. Cefoxitin resistance of stool recovered prior to bowel prep (left panel) and stool recovered on the day of surgery (right panel). **B.** Neomycin resistance of stool recovered prior to bowel prep (left panel) and stool recovered on the day of surgery (right panel). MIC=mean inhibitory concentration. NR=patients without development of postoperative recurrence; R=patients who develop a postoperative recurrence. P values were determined by unpaired two-tailed Mann–Whitney-test (A, B). Each dot represents one sample size, n=1.

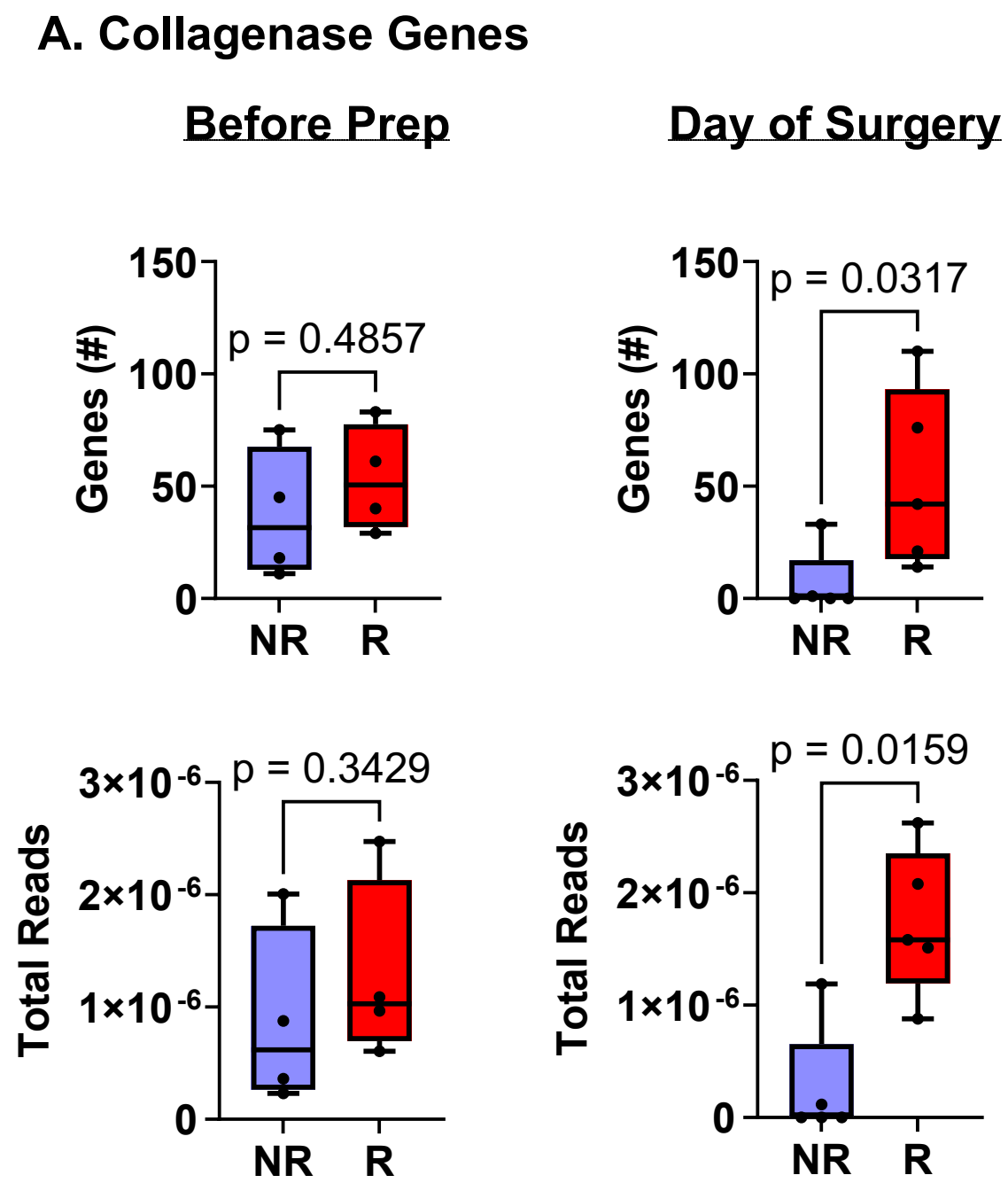

Supplementary Figure 3

A. Shotgun metagenomics on stool recovered prior to bowel prep and on the day of surgery showing the number of bacterial collagenase genes (top panel) and the total reads of bacterial collagenase genes. NR=patients without development of postoperative recurrence; R=patients who develop a postoperative recurrence. P values were determined by unpaired two-tailed Mann–Whitney-test. Each dot represents one sample size, n=1.

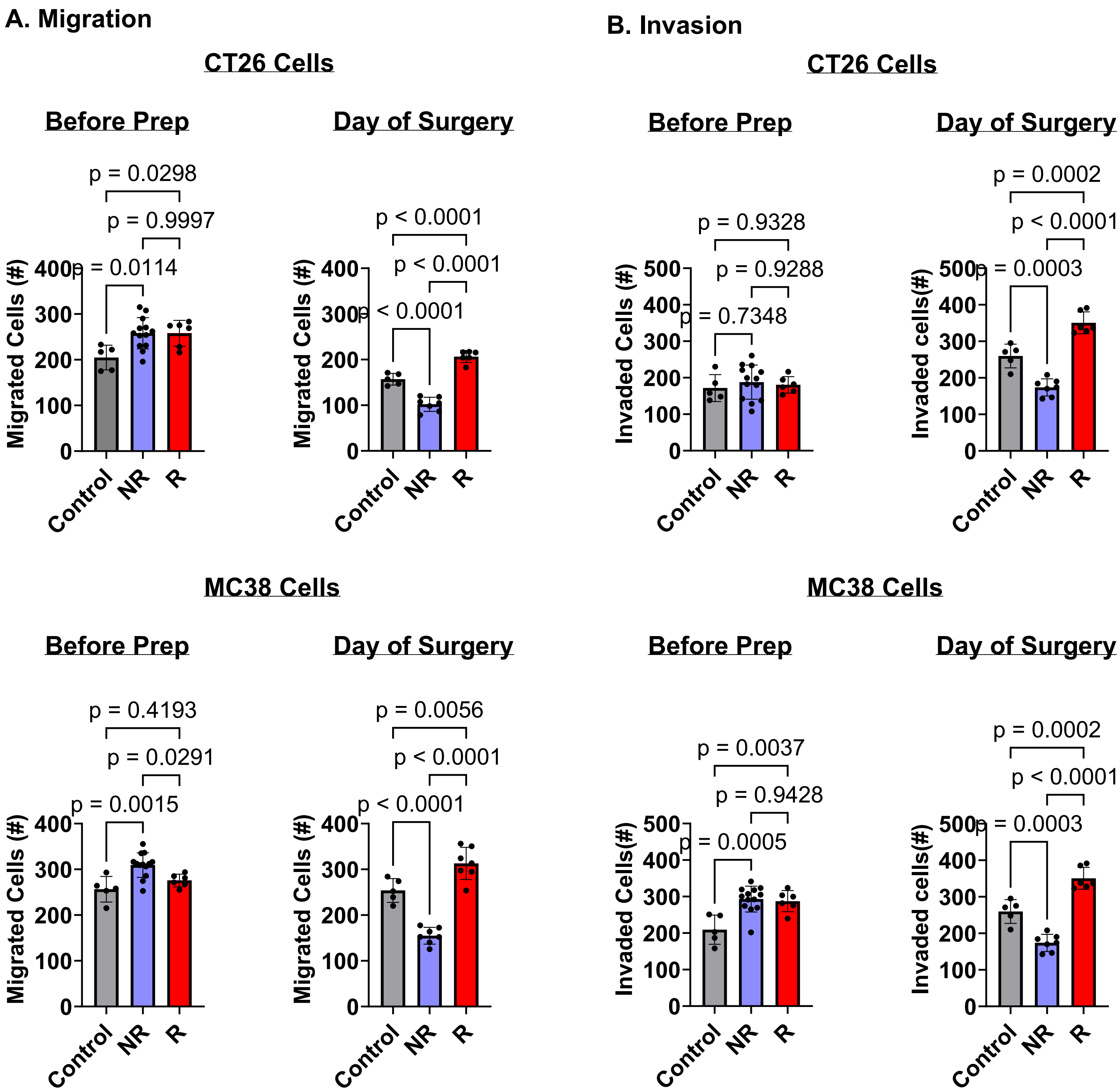

Supplementary Figure 4

A-B. The effect of bacterial cell-free supernatants on cancer cell migration (**A**) and invasion (**B**) of murine-derived CT26 and MC38 cancer cells. Error bars ± standard deviation. Solid line represents the median. NR=patients without development of postoperative recurrence; R=patients who develop a postoperative recurrence. P values were determined by one-way ANOVA with multiple comparison test. Each dot represents one sample size, n=1. Each dot represents the average value of minimum five fields of images for each sample. All experiments were repeated three times and were conducted as biologically independent experiments. Data are expressed as mean ± SD.

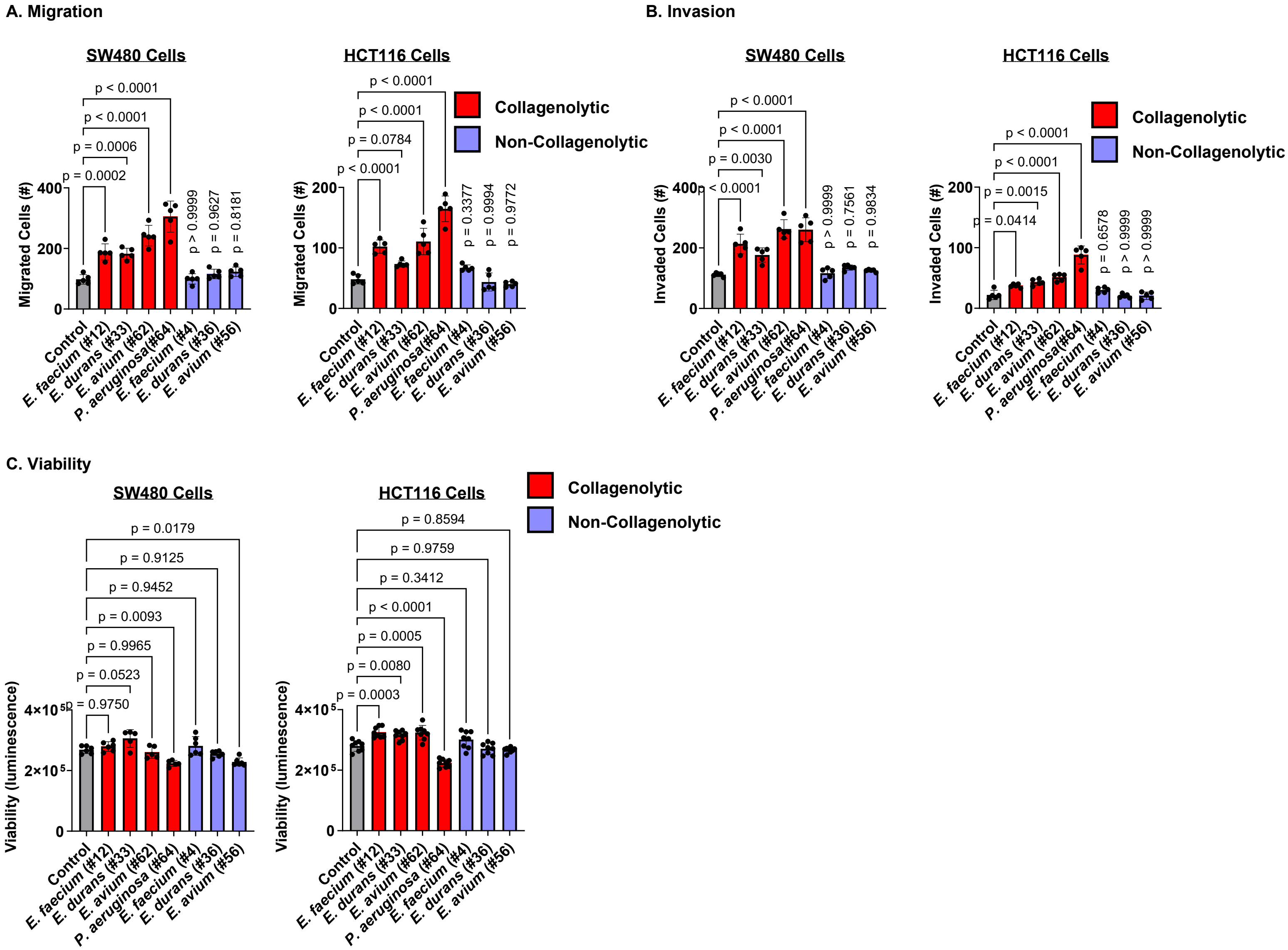

Supplementary Figure 5

The effect of bacterial cell-free supernatants from isolated strains on cancer cell migration (**A**), invasion (**B**), and proliferation (**C**) of human-derived SW480 and HCT116 cancer cells. Error bars ± standard deviation. Solid line represents the median. P-value is calculated by one-way ANOVA with multiple comparison test. P value of each strain compared to the control is presented. Each dot represents the average value of four fields of images (A, B) or the average of seven technical replicates (C). All experiments were repeated three times and were conducted as biologically independent experiments. Data are expressed as mean ± SD.

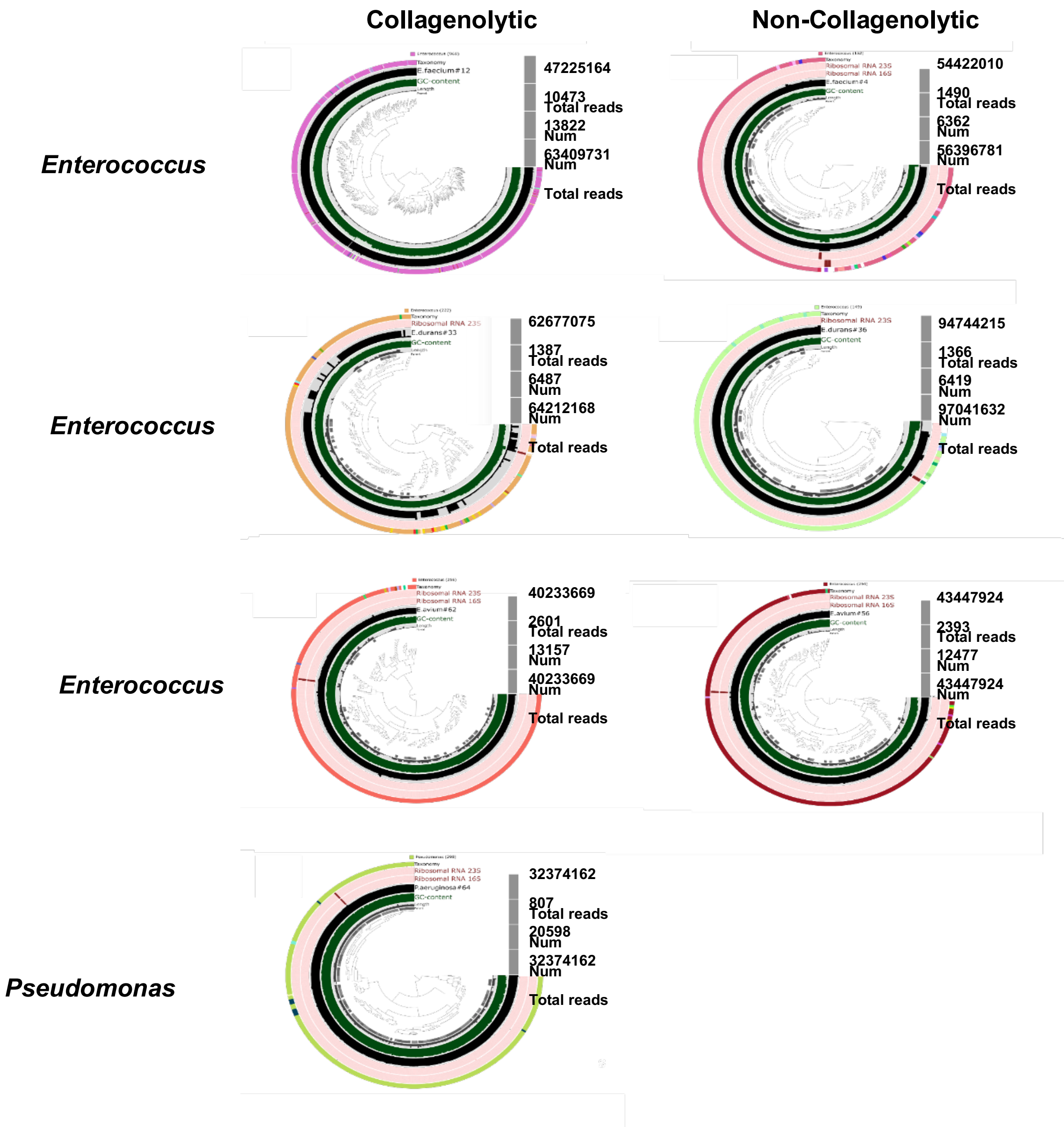

Supplementary Figure 6

Whole genome sequencing of the seven isolates that were used for *in vitro* testing. For *E. faecium*, *E. durans*, and *E. avium*, we cultivated and identified both collagenase-producing and non-collagenase-producing strains. Only collagenase-producing strains of *P. aeruginosa* were able to be identified.

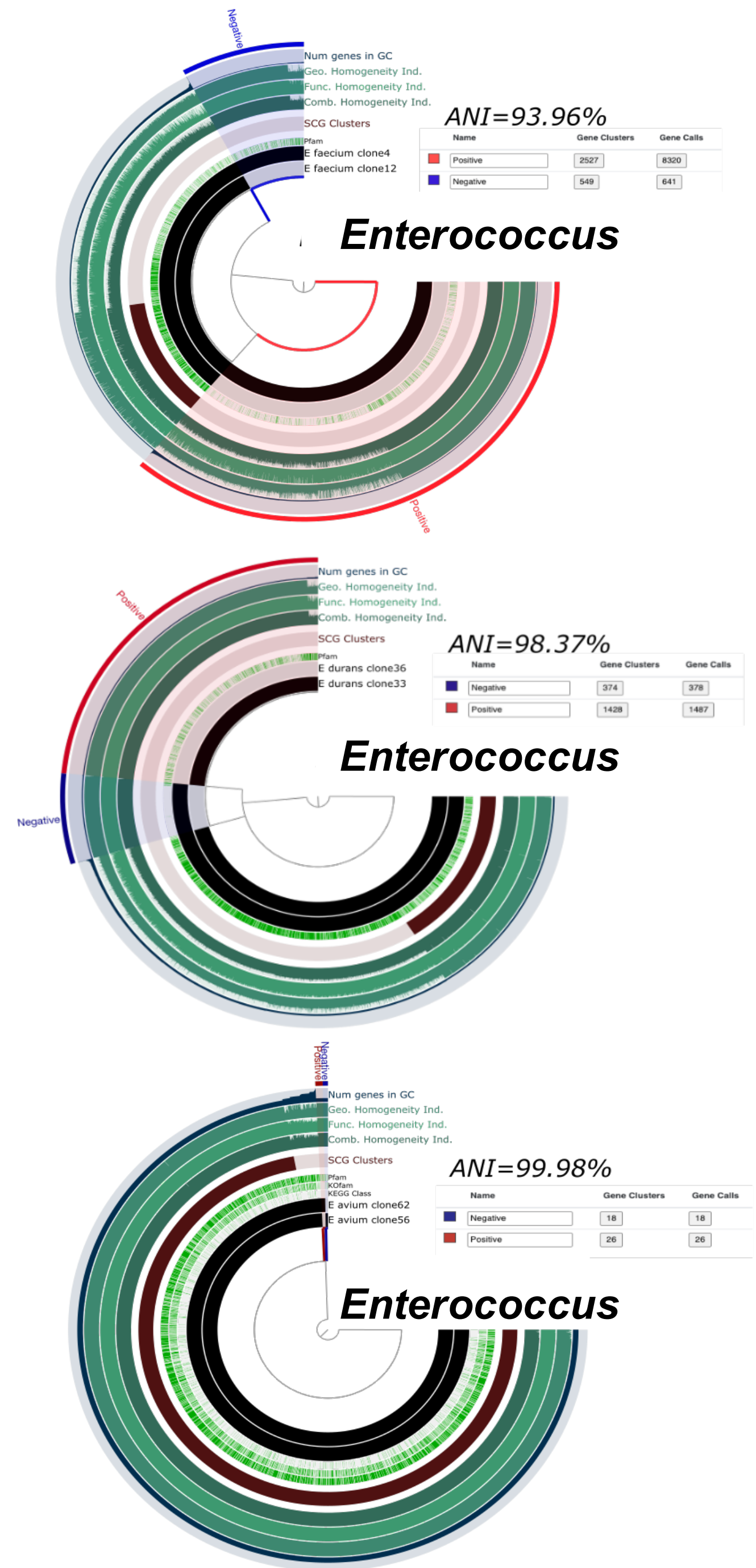

Supplementary Figure 7

Pangenomic analysis of each species, comparing the collagenase-producing and non-collagenase-producing strains. ANI = average nucleotide identity.

Supplementary Table 1: Patient Demographics

	Total (n=19)	Postoperative Recurrence No (n=13)	Postoperative Recurrence Yes (n=6)	P Value
Age, mean (SD)	64.9 (8.6)	64.8 (7.5)	65.3 (12.1)	0.95
Male, n (%)	11 (57.9)	8 (61.5)	3 (50)	0.47
BMI, mean (SD)	29.7 (7.1)	28.7 (6.3)	32 (9.5)	0.31
Race				
Caucasian, n (%)	13 (68)	8 (61.5)	5 (83.3)	0.60
AA, n (%)	6 (32)	5 (38.5)	1 (16.7)	
Antibiotic use, n (%)				
Last 90 days	10 (52.6)	7 (53.8)	3 (50)	0.87
Location, n (%)				
Colon	13 (68.4)	9 (69.2)	4 (66.7)	1.0
Rectum	6 (31.6)	4 (30.7)	2 (33.3)	
Tumor Grade, (%)				
1	2 (10.5)	2 (15.4)	0 (0)	1.0
2	15 (78.9)	10 (76.9)	5 (83.3)	
3	2 (10.5)	1 (7.7)	1 (16.7)	
Lymph Nodes Recovered, mean (SD)	16.9 (3.0)	17.1 (3.1)	16.6 (2.7)	.74
AJCC Clinical Stage$^{\alpha}$, n (%)				
I	4 (21.1)	4 (30.8)	0 (0)	0.46
II	7 (36.8)	4 (30.8)	3 (50)	
III	8 (42.1)	5 (38.5)	3 (50)	
AJCC Pathologic Stage$^{\beta}$, n (%)				
I	8 (42.1)	8 (61.5)	1 (16.7)	0.24
II	7 (36.8)	4 (30.8)	3 (50)	
III	4 (21.1)	2 (15.4)	2 (33.3)	
Chemotherapy/radiation, n (%)	12 (63.2)	8 (61.5)	4 (66.7)	0.99
Years follow-up, median (IQR)	2.0 (4.3)	5.1 (3.2)	.85 (.63)	

SD = standard deviation

BMI = body mass index

AA = African American

IQR = interquartile range

$^{\alpha}$Pathological stage if no neoadjuvant therapy; clinical stage if neoadjuvant therapy

$^{\beta}$Pathological stage for all patients

Comparison of the unpaired two-group was performed by Student's T-test. Comparison of proportions was performed using Fisher's exact tests

Supplementary Table 2: Bacterial Colonies Isolated from Recurrence Patients

		Collagenolytic Activity	
Colony ID	Bacteria Species	Agar Milk Plate (Y/N)	Liquid Culture (A.U. +/- SD)
1	Enterococcus faecium	N	$4.07x10^6 \pm 4.57x10^6$
2	Enterococcus faecium	N	$2.77x10^6 \pm 1.08x10^6$
3	Enterococcus faecium	N	$1.68x10^6 \pm 2.92x10^6$
4*	Enterococcus faecium	N	0
5	Enterococcus faecium	N	0
6	Enterococcus faecium	N	0
7	Enterococcus faecium	Y	$1.06x10^7 \pm 3.58x10^6$
8	Enterococcus faecium	N	$4.22x10^5 \pm 4.61x10^5$
9	Enterococcus faecium	N	$2.22x10^6 \pm 4.61x10^5$
10	Enterococcus faecium	N	$1.69x10^6 \pm 6.10x10^6$
11	Enterococcus faecium	N	0
12*	Enterococcus faecium	Y	$1.01x10^7 \pm 7.97x10^6$
13	Enterococcus faecium/durans	N	$1.59x10^6 \pm 6.37x10^5$
14	Enterococcus faecium	N	$2.00x10^6 \pm 9.93x10^5$
15	Enterococcus faecium/durans	N	$4.36x10^6 \pm 5.62x10^5$
16	Enterococcus faecium	N	$2.69x10^6 \pm 9.28x10^4$
17	Micrococcus luteus	Y	$5.49x10^6 \pm 1.11x10^6$
18	Micrococcus species/luteus	Y	$3.12x10^7 \pm 3.55x10^6$
19	Micrococcus species/luteus	N	0
20	Micrococcus species/lactis/yunnanesis	Y	0
21	Micrococcus species/luteus	Y	$1.91x10^7 \pm 2.78x10^7$
22	Micrococcus species/luteus/yunnanensis	Y	$3.77x10^6 \pm 2.46x10^6$
23	Micrococcus species/luteus	N	0
24	Micrococcus species/luteus/yunnanensis	N	0
25	Micrococcus species/luteus	Y	0
26	Micrococcus species/luteus	N	0
27	Micrococcus species/luteus	N	0
28	Micrococcus luteus	Y	$5.37x10^6 \pm 1.85x10^6$
29	Enterococcus durans	N	$3.98x10^6 \pm 4.37x10^6$
30	Enterococcus durans	N	$2.31x10^6 \pm 1.59x10^6$
31	Enterococcus durans	N	$6.54x10^6 \pm 3.82x10^6$
32	Enterococcus durans	N	$1.10x10^6 \pm 2.21x10^6$
33*	Enterococcus durans	Y	$1.09x10^7 \pm 3.41x10^6$
34	Enterococcus durans	N	$1.67x10^6 \pm 1.64x10^6$
35	Enterococcus durans	N	0
36*	Enterococcus durans	N	0
37	Enterococcus durans	N	$2.11x10^6 \pm 4.65x10^5$

38	Enterococcus durans	N	0
39	Weisella cibaria	N	0
40	Weisella cibaria/confusa, Leuconostic lactis	N	0
41	Weisella cibaria/confusa, Leuconostic lactis	N	$1.42 \times 10^{6} \pm 3.25 \times 10^{6}$
42	Weisella cibaria/confusa, Leuconostic lactis	N	$7.04 \times 10^{6} \pm 3.69 \times 10^{6}$
43	Weisella cibaria/confusa, Leuconostic lactis	N	$3.26 \times 10^{6} \pm 8.60 \times 10^{6}$
44	Weisella cibaria/confusa,Leuconostic lactis,Limocillolactobacillus fermentum	N	$4.99 \times 10^{6} \pm 1.08 \times 10^{7}$
45	Weisella cibaria/confusa,Leuconostic lactis,Limocillolactobacillus fermentum	N	$1.09 \times 10^{7} \pm 1.76 \times 10^{6}$
46	Weisella cibaria/confusa,Leuconostic lactis,Limocillolactobacillus fermentum	N	$2.09 \times 10^{6} \pm 6.74 \times 10^{6}$
47	Weisella cibaria/confusa,Leuconostic lactis,Limocillolactobacillus fermentum	N	$1.67 \times 10^{7} \pm 5.73 \times 10^{6}$
48	Escherichia coli	N	0
49	Escherichia coli	N	0
50	Escherichia coli	N	0
51	Escherichia coli	N	0
52	Escherichia coli	N	0
53	Escherichia coli	N	0
54	Escherichia coli	N	0
55	Escherichia coli	N	0
56*	Enterococcus avium	N	0
57	Enterococcus avium	N	0
58	Enterococcus avium	Y	0
59	Enterococcus avium	N	0
60	Enterococcus avium	N	0
61	Enterococcus avium/gilvus	N	$6.85 \times 10^{5} \pm 3.97 \times 10^{5}$
62*	Enterococcus avium	Y	$3.01 \times 10^{6} \pm 2.22 \times 10^{6}$
63	Pseudomonas aeruginosa	Y	$4.48 \times 10^{7} \pm 1.23 \times 10^{7}$
64*	Pseudomonas aeruginosa	Y	$9.73 \times 10^{7} \pm 3.83 \times 10^{7}$
65	Pseudomonas aeruginosa	Y	$3.22 \times 10^{7} \pm 1.16 \times 10^{7}$
66	Pseudomonas aeruginosa	Y	$1.06 \times 10^{7} \pm 4.68 \times 10^{6}$
67	Pseudomonas aeruginosa	Y	$7.49 \times 10^{6} \pm 4.88 \times 10^{6}$
68	Pseudomonas aeruginosa	Y	$1.42 \times 10^{7} \pm 8.23 \times 10^{6}$
69	Staphylococcus species	N	$1.13 \times 10^{6} \pm 7.47 \times 10^{6}$
70	Microbacterium paraoxydans	Y	$1.20 \times 10^{7} \pm 2.54 \times 10^{7}$
71	Bacillus cereus	Y	$4.91 \times 10^{7} \pm 3.12 \times 10^{7}$
72	Streptococcus mutans	N	$8.01 \times 10^{5} \pm 8.89 \times 10^{6}$

*Colonies selected for in vitro experiments and whole genome sequencing.

Value for liquid culture collagenolytic activity is luminescence normalized to OD (600nm) (A.U. = arbitrary unit).

Supplementary Table 3: STORMS Checklist

Number	Item	Recommendation	Item Source	Yes/No/NA	Comments or location in manuscript
1.0	Structured or Unstructured Abstract	Abstract including information on background, methods, results, and conclusions is presented in unstructured format.	STORMS		2
1.1	Study Design	State study design in abstract.	STORMS	NA	
1.2	Sequencing methods	State the strategy used for metagenomic classification.	STORMS	NA	
1.3	Specimens	Describe body site(s) studied.	STORMS	NA	
2.0	Background and Rationale	Background, scientific evidence and the study objectives were described.	STORMS		3
2.1	Hypotheses	State the pre-specified hypothesis. If the study is exploratory, state any pre-specified study objectives.	STORMS	NA	
3.0	Study Design	Prospective cohort study.	STORMS		17
3.1	Participants	Patients aged ≥ 18 years old undergoing an elective colorectal resection for malignancy at the University of Chicago Comprehensive Cancer Center were recruited to participate in this study.	STORMS		17
3.2	Geographic location		MIxS: geographic location (country and/or sea,region)	NA	
3.3	Relevant Dates	Patients were followed for five years postoperatively	STORMS		17

3.4	Eligibility criteria	Patients aged ≥ 18 years old undergoing an elective colorectal resection for malignancy at the University of Chicago Comprehensive Cancer Center were recruited. Patients were excluded if they currently had an ostomy or had a contraindication to receiving a combined bowel preparation.	Modified STROBE		17
3.5	Antibiotics Usage	All patients had a mechanical purgative combined with oral neomycin and oral metronidazole the day before surgery and received intravenous cefoxitin on the day of surgery prior to surgical incision.	STORMS		17
3.6	Analytic sample size	Explain how the final analytic sample size was calculated, including the number of cases and controls if relevant, and reasons for dropout at each stage of the study. This should include the number of individuals in whom microbiome sequencing was attempted and the number in whom microbiome sequencing was successful.	STORMS		N/A – no dropouts
3.7	Longitudinal Studies	Patients were followed for five years postoperatively.	STORMS		17
3.8	Matching	Participants were individual	Modified STROBE		17
3.9	Ethics	Approved by the University of Chicago Institutional Review Board (IRB 17-0417).	STORMS		17
4.0	Laboratory methods	All stool samples were processed in a designated and certified fume hood.	STORMS		18
4.1	Specimen collection	The 'before bowel prep' samples were collected using the BioCollector stool kit (BioCollective). The 'day of surgery' sample was collected from the surgically resected specimen.	MIxS: sample collection device or		17

			method; host body site		
4.2	Shipping	The 'before bowel prep' samples were shipped overnight via FedEx by patients using pre-shipped stool collection kit.	STORMS		17
4.3	Storage	All samples were processed immediately, and aliquots of homogenized stool were stored either alone or mixed with 20% glycerol at -80ºC until they were used for future experiments.	STORMS		18
4.4	DNA extraction	QIAamp PowerFecal Pro DNA Kit (Qiagen)	MIxS: nucleic acid extraction		17
4.5	Human DNA sequence depletion or microbial DNA enrichment	Describe whether human DNA sequence depletion or enrichment of microbial or viral DNA was performed.	STORMS	NA	
4.6	Primer selection	V4-V5 region of the 16S rRNA gene was PCR amplified with primers (Fwd-AYTGGGYDTAAAGNG) and (Rev-CCGTCAATTYHTTTRAGT	MIxS: pcr primers		18
4.7	Positive Controls		STORMS	NA	
4.8	Negative Controls		STORMS	NA	
4.9	Contaminant mitigation and identification	All reagents were either autoclaved or filtered using 0.20 µm filter	STORMS		21
4.10	Replication		STORMS	NA	

4.11	Sequencing strategy	16S rRNA (V4-V5 region), Shotgun and whole genome sequencing were used.	MIxS: sequencing method		17-18
4.12	Sequencing methods	16S rRNA and whole genome sequences were sequenced using the Illumina MiSeq<br>Shot sequencing was done using Illumina NextSeq 500.	STORMS		19-21
4.13	Batch effects	Detail any blocking or randomization used in study design to avoid confounding of batches with exposures or outcomes. Discuss any likely sources of batch effects, if known.	STORMS	NA	
4.14	Metatranscriptomics		STORMS	NA	
4.15	Metaproteomics		STORMS	NA	
4.16	Metabolomics		STORMS	NA	
5.0	Data sources/ measurement		MIxS: host disease status	NA	
6.0	Research design for causal inference		STORMS	NA	
6.1	Selection bias	Patients were excluded if they currently had an ostomy or had a contraindication to receiving a combined bowel preparation.	STORMS		17
7.0	Bioinformatic and Statistical Methods	Qiime diversity core-metrics, kneaddata Metaphlan4, Trimmomatic, Anvi'o, Prodigal, Kyoto Encyclopedia of Genes and Genomes (KEGG) and anvi-run-pfams for bioinformatics and<br>GraphPad Prism for statistical analysis	STORMS		19-20, 24

7.1	Quality Control	Illumina-compatible libraries were generated using the Qiagen QIASeq 1-step amplicon kit and sequenced on the Illumina MiSeq platform generating 2×250 PE reads and >10,000 reads per sample and analyzed on the Qiime2 platform (v2024.5). Briefly FASTQs were demultiplexed and quality filtered via the DADA2 algorithm with truncation of all reads to 190bp to generate amplicon sequence variant (ASV) counts and representative sequences	MIxS: sequence quality check		19
7.2	Sequence analysis	Taxonomic classification was performed using a Bayesian RDP classifier trained on 16S sequences from the SILVA database (v138_2).	MIxS: feature prediction; similarity search method		19
7.3	Statistical methods	Statistical analyses were performed using GraphPad Prism software (version 10.5.0) for antibiotics, collagenase activity, invasion, and migration assays. Comparison of unpaired groups was performed using Mann-Whitney test or Wilson signed rank for paired groups. Comparison of proportions was performed using Fisher's exact tests. Wilcoxon Rank Sum tests were used for bacterial alpha diversity, and permutational multivariate analysis of variance (PERMANOVA) and Kruskal-Wallis tests were used for bacterial beta diversity. An “exact” logistic regression for the dichotomized outcome of whether a recurrence occurred or did not occur was conducted[15]. P values ≥0.05 were considered statistically significant.	Modified STROBE		24
7.4	Longitudinal analysis	Longitudinal analysis was performed comparing each patients pre-bowel prep sample to their day of surgery sample. Change in the microbiome due to bowel prep was quantified for each patient as the unweighted UniFrac	STORMS		19-20

		distance or PC distance between the day of surgery sample and before bowel prep sample			
7.5	Subgroup analysis		STROBE	NA	
7.6	Missing data		STROBE	NA	
7.7	Sensitivity analyses		STROBE	NA	
7.8	Findings		STORMS	NA	
7.9	Software	Qiime diversity core-metrics, kneaddata Metaphlan4, Trimmomatic, Anvi'o, Prodigal, Kyoto Encyclopedia of Genes and Genomes (KEGG) and anvi-run-pfams	Modified STREGA		19-20
8.0	Reproducible research	Expect reproducible data by others in a larger cohort study	STORMS		
8.1	Raw data access	The 16S rRNA, shotgun metagenomics, and whole genome sequencing data generated in this study have been deposited in the NCBI Sequence Read Archive (SRA) under project number PRJNA1233323. All other data generated in this study have been deposited in Zenodo under DOU: 10.5281/zenodo.19361145 (zenodo.org).	STORMS		24-25
8.2	Processed data access	All data is provided in this manuscript or in supplementary information	STORMS		24-25
8.3	Participant data access	Metadata for the microbiome analysis is publicly available as part of the NCBI Sequence Read Archive (SRA) under project number PRJNA1233323 or is available by request of the corresponding author.	STORMS		24-25

8.4	Source code access		STORMS	NA	
8.5	Full results		STORMS	NA	
9.0	Descriptive data	Information of patients' demographic and clinical stage is Reported in supplementary information as a supplementary table 1.	STROBE		
10.0	Microbiome data	Patients who develop postoperative recurrence have distinct microbiome signatures.	STORMS		5-8
10.1	Taxonomy	Identify taxonomy using standardized taxon classifications that are sufficient to uniquely identify taxa.	STORMS		Throughout the manuscript
10.2	Differential abundance		STORMS	NA	
10.3	Other data types	.	STORMS	NA	
10.4	Other statistical analysis		STORMS	NA	
11.0	Key results	This study provides evidence that enrichment of the perioperative gut-microbiome with collagenolytic bacteria that can promote a migratory and invasive cancer cell phenotype that is highly associated with the development of postoperative tumors.	STROBE		13
12.0	Interpretation	This study shows a potential link between bowel prep-resistant collagenolytic microbiota and the development of post-operative CRC recurrence in human patients.	STROBE		15

13.0	Limitations	Sample size is small	STROBE		15
13.1	Bias	Potential of change of microbiome composition during in vitro culture	STORMS		15
13.2	Generalizability	Multi-institutional level study would be great.	STROBE		15
14.0	Ongoing/future work	Validation in a larger cohort	STORMS		13
15.0	Funding	BDS: Cancer Research Foundation Young Investigator Award; NIH NCI 1K08CA248957-01A1.	STROBE		30
15.1	Acknowledgements	We thank Huaiying Lin, Nicholas P. Dylla, and Eric Palmer at the Duchossois Family Institute for their help in processing the metagenomic sequencing.	STORMS		30
15.2	Conflicts of Interest	All authors report no conflicts of interest.	STORMS		30
16.0	Supplements	All data is publicly available.	STORMS		24-25
17.0	Supplementary data	All supplementary data is provided in supplemental information and deposited in NCBI Sequence Read Archive (SRA) under project number PRJNA1233323 and Zenodo under DOI: 10.5281/zenodo.20274431(zenodo.org).	STORMS		24-25
