# Supplementary material for: A virulent bacterial signature is associated with the development of recurrence following colorectal cancer surgery
Source: Nat Commun. 2026 Jul 6;17:8361. doi: 10.1038/s41467-026-74889-x (PMC13473480; doi:10.1038/s41467-026-74889-x)
Supplement: Supplementary file 2 [file 41467_2026_74889_MOESM2_ESM.pdf]

nature portfolio

Corresponding author(s): Benjamin Shogan

Last updated by author(s): 4.2.2026

Reporting Summary

Nature Portfolio wishes to improve the reproducibility of the work that we publish. This form provides structure for consistency and transparency in reporting. For further information on Nature Portfolio policies, see our Editorial Policies and the Editorial Policy Checklist.

Statistics

For all statistical analyses, confirm that the following items are present in the figure legend, table legend, main text, or Methods section.

n/a	Confirmed	
☐	☒	The exact sample size (*n*) for each experimental group/condition, given as a discrete number and unit of measurement
☐	☒	A statement on whether measurements were taken from distinct samples or whether the same sample was measured repeatedly
☐	☒	The statistical test(s) used AND whether they are one- or two-sided<br>*Only common tests should be described solely by name; describe more complex techniques in the Methods section.*
☐	☒	A description of all covariates tested
☒	☐	A description of any assumptions or corrections, such as tests of normality and adjustment for multiple comparisons
☐	☒	A full description of the statistical parameters including central tendency (e.g. means) or other basic estimates (e.g. regression coefficient) AND variation (e.g. standard deviation) or associated estimates of uncertainty (e.g. confidence intervals)
☐	☒	For null hypothesis testing, the test statistic (e.g. *F*, *t*, *r*) with confidence intervals, effect sizes, degrees of freedom and *P* value noted<br>*Give P values as exact values whenever suitable.*
☒	☐	For Bayesian analysis, information on the choice of priors and Markov chain Monte Carlo settings
☒	☐	For hierarchical and complex designs, identification of the appropriate level for tests and full reporting of outcomes
☒	☐	Estimates of effect sizes (e.g. Cohen's *d*, Pearson's *r*), indicating how they were calculated

Our web collection on statistics for biologists contains articles on many of the points above.

Software and code

Policy information about availability of computer code

Data collection	No software was used.
Data analysis	Statistical analyses were performed using GraphPad Prism software (version 10.5.0). Shotgun metagenomics: Libraries were sequenced on an Illumina NextSeq 500 to generate 2x150 reads. Adapters were trimmed off the raw reads with low quality bases (PHRED<30) with Trimmomatic v.0.39. Taxonomic profiling was performed using Metaphlan428. Single-sample assemblies were generated using MEGAHIT v1.2.9, with resulting contigs imported and analyzed in the Anvi'o platform (v8).

For manuscripts utilizing custom algorithms or software that are central to the research but not yet described in published literature, software must be made available to editors and reviewers. We strongly encourage code deposition in a community repository (e.g. GitHub). See the Nature Portfolio guidelines for submitting code & software for further information.

Data

Policy information about availability of data

All manuscripts must include a data availability statement. This statement should provide the following information, where applicable:

- Accession codes, unique identifiers, or web links for publicly available datasets
- A description of any restrictions on data availability
- For clinical datasets or third party data, please ensure that the statement adheres to our policy

Data availability
All data is publicly available. The 16S rRNA, shotgun metagenomics, and whole genome sequencing data generated in this study have been deposited in the NCBI

Sequence Read Archive (SRA) under project number PRJNA1233323. All other data generated in this study have been deposited in Zenodo under DOU: 10.5281/zenodo.19361145 (zenodo.org). All other data supporting this study are available at or is present within the manuscript.

Research involving human participants, their data, or biological material

Policy information about studies with human participants or human data. See also policy information about sex, gender (identity/presentation), and sexual orientation and race, ethnicity and racism.

Reporting on sex and gender	We collected 'sex' as a categorization variable. This was only used to describe the patient cohort. Our findings do not support relating to only one sex.
Reporting on race, ethnicity, or other socially relevant groupings	We collected 'race' as a categorization variable. This was only used to describe the patient cohort. This was self-reported by each subject. This variable was not considered in the analysis.
Population characteristics	The average age was 64.9 years (SD 8.6) and 58% were male. All patients had a diagnosis of colorectal adenocarcinoma with 68.4% being in the colon and 31.6% in the rectum. AJCC stage was stage 1 (21.1%), 2 (47.4%), 3 (31.6%)/
Recruitment	Consecutive patients aged ≥ 18 years old undergoing an elective colorectal resection for malignancy at the University of Chicago Comprehensive Cancer Center were recruited to participate in this study.
Ethics oversight	University of Chicago IRB #17-0417

Note that full information on the approval of the study protocol must also be provided in the manuscript.

Field-specific reporting

Please select the one below that is the best fit for your research. If you are not sure, read the appropriate sections before making your selection.

[x] Life sciences [ ] Behavioural & social sciences [ ] Ecological, evolutionary & environmental sciences

For a reference copy of the document with all sections, see nature.com/documents/nr-reporting-summary-flat.pdf

Life sciences study design

All studies must disclose on these points even when the disclosure is negative.

Sample size	This was a preliminary study of 19 consecutive patients undergoing surgery for colorectal malignancy at the University of Chicago Comprehensive Cancer Center and therefore there was no predetermined power analysis.
Data exclusions	No data was excluded.
Replication	All in vitro assays were replicated at least twice to confirm reproducibility. When running each individual experiment the assays were done in biological triplicate.
Randomization	There was no intervention and therefore no randomization of subjects.
Blinding	Sequencing and metagenomics data creation/analysis was blinded to the development of the primary outcome (recurrence).

Reporting for specific materials, systems and methods

We require information from authors about some types of materials, experimental systems and methods used in many studies. Here, indicate whether each material, system or method listed is relevant to your study. If you are not sure if a list item applies to your research, read the appropriate section before selecting a response.

Materials & experimental systems

n/a	Involved in the study	
[x]	[ ]	Antibodies
[ ]	[x]	Eukaryotic cell lines
[x]	[ ]	Palaeontology and archaeology
[x]	[ ]	Animals and other organisms
[ ]	[x]	Clinical data
[x]	[ ]	Dual use research of concern
[x]	[ ]	Plants

Methods

n/a	Involved in the study	
[x]	[ ]	ChIP-seq
[x]	[ ]	Flow cytometry
[x]	[ ]	MRI-based neuroimaging

Eukaryotic cell lines

Policy information about cell lines and Sex and Gender in Research

Cell line source(s)	We purchased CT26 from ATCC(ATCC CRL-2638) and MC38 from kerafast. HCT116 and SW480 were provided by Dr. Christopher Weber at the University of Chicago.
Authentication	When the cells were received they contained an authentication certificate.
Mycoplasma contamination	All cells were tested for mycoplasma contamination and they were negative.
Commonly misidentified lines (See ICLAC register)	N/A

Clinical data

Policy information about clinical studies

All manuscripts should comply with the ICMJE guidelines for publication of clinical research and a completed CONSORT checklist must be included with all submissions.

Clinical trial registration	This study used biospecimens but was not part of a clinical trial and there is no clinical trial registration.
Study protocol	The details used for the collection of the specimens are detailed in methods section of the manuscript.
Data collection	Stool samples were collected before and after the patient completed the combined bowel preparation. Pre-bowel preparation samples were collected using the BioCollector stool kit (BioCollective). Post-bowel preparation samples were collected from the resected intestinal segment in the pathology laboratory on the day of surgery. All samples were processed immediately by homogenizing them in lysing matrix D (MP Biomedicals, 6913100). Aliquots of homogenized stool were stored either alone or with 20% glycerol at -80oC until it was used for future experiments.
Outcomes	The primary outcome measure was the development of postoperative recurrence. Patients are followed for five years postoperatively at the University of Chicago Comprehensive Cancer Center for the development of recurrence using the guidelines published by the National Comprehensive Cancer Network (NCCN). The electronic medical record was reviewed for each subject and the development of recurrence was determined if the patients oncologist documented recurrence.

Plants

Seed stocks	*Report on the source of all seed stocks or other plant material used. If applicable, state the seed stock centre and catalogue number. If plant specimens were collected from the field, describe the collection location, date and sampling procedures.*
Novel plant genotypes	*Describe the methods by which all novel plant genotypes were produced. This includes those generated by transgenic approaches, gene editing, chemical/radiation-based mutagenesis and hybridization. For transgenic lines, describe the transformation method, the number of independent lines analyzed and the generation upon which experiments were performed. For gene-edited lines, describe the editor used, the endogenous sequence targeted for editing, the targeting guide RNA sequence (if applicable) and how the editor was applied.*
Authentication	*Describe any authentication procedures for each seed stock used or novel genotype generated. Describe any experiments used to assess the effect of a mutation and, where applicable, how potential secondary effects (e.g. second site T-DNA insertions, mosiacism, off-target gene editing) were examined.*
