# Supplementary material for: A virulent bacterial signature is associated with the development of recurrence following colorectal cancer surgery
Source: Nat Commun. 2026 Jul 6;17:8361. doi: 10.1038/s41467-026-74889-x (PMC13473480; doi:10.1038/s41467-026-74889-x)
Supplement: Supplementary file 3 [file 41467_2026_74889_MOESM3_ESM.pdf]

nature portfolio

Peer Review File

A virulent bacterial signature is associated with the development of recurrence following colorectal cancer surgery

Corresponding Author: Dr Benjamin Shogan

This manuscript has been previously reviewed at another journal. This document only contains information relating to versions considered at Nature Communications.

This file contains all reviewer reports in order by version, followed by all author rebuttals in order by version.

Version 0:

Reviewer comments:

Reviewer #1

(Remarks to the Author)
Drs. Shogan and authors have submitted a revision of their initial report on a 'Virulent and antibiotic-resistant bacterial signature associated with the development of postoperative recurrence after colorectal cancer surgery'.

I have noted my comments below. In general I am enthusiastic about these data and am satisfied with the revisions and considerations made to the original submitted work.

Noteworthy findings:
a) unique signature in stool collected prior to surgery
b) unique signature on day of surgery stool (recurrence vs. no recurrence)
c) persistence of a signature (pre and day of surgery) - this is important
d) bacteria 23x > in stool on day of surgery for those with recurrence vs. those without (16srRNA)
- notably bowel prep appeared to not impact the bacterial load in those with recurrence
e) implication of resistance mechanisms from the stool of those with recurrence with some interesting caveats/limitations of the findings (bottom line --> abx resistance is associated with recurrent disease).
f) more collagenytic activity in those with recurrence than without and association with oncologic outcome - all in all, abx sensitivity and collagenase activity showed periop bowel prep uncovering an abx-resistant collagenytic microbiome significantly and independently associated with recurrent disease
g). proof of principle co-incubation studies with supernatatant from bacteria collected from day of surgery recurrent patients (collagenolytic) and cancer cell lines increased migration and invasion vs. non-collagenolytic bacteria from non-recurrent patients (no difference between recurrent and non-recurrent patients when non-collagenolytic pre-bowel prep samples were used --> periop bowel prep showed the presence of a pro-metastatic microbiome in those that developed recurrence).

Findings significant to the field?:
Yes and stands apart from the literature given the prospective nature of the work and potential for future validation and insight into mechanisms of resistance and the interplay between the microbiome and recurrent disease.

Does the work support conclusions and claims: yes - adequate evidence provided

Are there flaws?: the limitations are acknowledged by the authors and honestly noted

Methodology: the study is sound and meets basic scientific standards

Is there adequate detail for reproduction?: Yes

Data and study are presented well - no major concerns with the figures or data presented

Reviewer #2

(Remarks to the Author)
I thank the authors for their response to my prior concerns. While I appreciate their efforts, my core concerns around sample size, statistical robustness, causality, and mechanistic depth remain. Below I outline my major concerns in more detail:

1) Interpretation and Claims:
-Many of the conclusions continue to extend beyond what the data support. For example, references to a “pro-metastatic collagenolytic microbiota” or statements such as “the in vitro analysis demonstrates that the collagenolytic microbiota… promotes tumor progression” (lines 269–270) are not substantiated. The authors tested uncharacterized supernatants from mixed fecal cultures on 2D cell lines, not defined taxa or in vivo tumors. These experiments can only be interpreted as cell line phenotypic changes, not tumor progression.
-The repeated use of terms like “pro-metastatic collagenolytic microbiome” should be tempered unless supported by experiments using defined, patient-derived isolates with demonstrated collagenolytic activity.

2) Reproducibility and Specificity:
-As I noted previously, a central weakness is the reliance on uncharacterized fecal supernatants. Without identifying specific bacterial taxa or quantifying their contributions, the results cannot be independently reproduced or validated.
-I strongly encourage the authors to sequence protease-positive colonies from their skim-milk agar assays via colony PCR + Sanger sequencing + BLAST to ID followed by WGS (very reasonable now, only $50-65) of isolates of interest and then test defined isolates in functional assays. This would provide a reproducible framework and allow others to confirm these findings and enormously strengthen the manuscript.

3) Data Clarification and Normalization:
-Bacterial load (Fig 1E): Please clarify whether 16S copy numbers were normalized per gram of stool. Post–bowel prep, the absolute mass of colon contents is drastically reduced, and without normalization these comparisons are misleading.
-Collagenase activity (Fig 2B): One NR sample shows measurable activity. Was stool actually obtained, and was bowel prep complete? How was input normalized for the broth assays (e.g., equal CFUs)? Could higher inoculum in NR samples yield activity comparable to R? These clarifications are important to assess whether differences reflect true biology or just variation in input.
-Migration/invasion assays (Fig 2E–G): How were supernatants standardized? Without normalization to bacterial load, CFU, or biomass, it is unclear whether the observed effects are due to collagenase or other factors in the mix.

4) Inconsistencies and Errors:
-Lines 229–231 appear contradictory: the text states there was no difference in collagenolytic activity on the day of surgery, yet Fig. 2B suggests otherwise. Please clarify.
The manuscript reports no significant global difference in pre–bowel prep microbiome composition or collagenase activity between recurrence and non-recurrence groups, yet the discussion refers to a “bowel-prep resistant, antibiotic-resistant collagenase bacterial signature.” It is unclear where this signature is arising from if it is not evident in the pre–bowel prep samples. In their response to my earlier concern about the timeline to metastasis, the authors acknowledged that they were not suggesting that bacteria present at the time of surgery were driving recurrence. Instead, they now hypothesize that the bowel prep “uncovered” a collagenolytic, pro-tumor bacterial community that must have been present beforehand and interacted with the primary tumor to drive micrometastatic disease prior to surgery. This interpretation seems inconsistent with their own data showing no pre–bowel prep differences and raises questions about whether the “bowel-prep resistant collagenolytic signature” is truly causal, or whether it is a correlate of recurrence identified only after manipulation by the bowel prep itself.

5) Relation to Prior Work:
-Given the authors’ prior findings on Enterococcus faecalis, was this taxon enriched in recurrence cases pre- or post–bowel prep? This analysis would provide a mechanistic bridge between the current work and earlier studies.

Overall, this study presents provocative preliminary data, but its impact is limited by small cohort size, reliance on uncharacterized supernatants, and over-interpretation of in vitro assays. Tempering the claims, clarifying methods (in particular normalization approaches), and moving toward taxon-level resolution will be critical to strengthen the scientific contribution and ensure reproducibility or even the ability for the community to try and reproduce the in vitro data, which is a major concern.

Reviewer #3

(Remarks to the Author)
In this manuscript, the authors have focused on the role of bowel-prep-resistant collagenolytic bacteria that are predicted to promote post-operative recurrence. Although these are interesting findings, the cohort size is simply too small and other clinical factors are missing that can have an impact on recurrence. Examples of a few are the molecular profiles (genetic mutations) of the tumors, grades of the tumor, number of lymph nodes removed, CEA levels, ctDNA status of the blood. I think the best way to conduct the study is to create a repository and wait until many more patients are analyzed, collecting many more clinical datapoints, and if a pattern is found and enough patients are enrolled, potentially performing either subgroup analyses, preferably utilizing propensity score matching (PSM). This is not to say it is not an important finding. It is

certainly worth reporting, but the authors need to state it is as such rather than making a statement that suggests such strong clinical relevance. The findings show an association that may have a clinical impact, depending on much larger studies, and hopefully through the formation of a post-surgical microbial repository.

Version 1:

Reviewer comments:

Reviewer #2

(Remarks to the Author)
I thank the authors for adding substantial new data and revising the text to address my previous concerns and comments. In particular, the identification and experimental validation of specific collagenic and non-collagenic strains significantly enhance the strength of the study and its conclusions. I have no further concerns.

Reviewer #3

(Remarks to the Author)
I remain concerned about the sampling strategy. In practice, stool collection from a freshly resected colorectal specimen is methodologically challenging and potentially variable, and additional procedural detail would be important. Clarification is needed regarding timing from resection to collection, the exact sampling site relative to the tumor and margins, whether the lumen was irrigated or flushed before sampling, and how manipulation or operative contamination was minimized. The “day of surgery” specimen—residual stool in direct contact with the tumor—sits at the center of the manuscript’s conclusions, yet without detailed standardization it is difficult to distinguish a true biological signal from procedural variability. More broadly, because this sample is tumor-adjacent, it remains unclear whether the signature reflects a causal microbial phenotype or tumor-associated selection in biologically aggressive cancers - or whether it confers aggressiveness. The absence of tumor tissue or mucosa-associated microbiome analysis further limits mechanistic inference and leaves the driver-versus-correlate question unresolved.

The small sample size (n=19; 6 recurrences) is the dominant limitation and materially constrains interpretability. Signals in Supplementary Table 1—such as tumor location (biofilm potential), recent antibiotic exposure, and stage distribution (two-thirds localized overall vs roughly half among recurrences)—raise the possibility of confounding that is likely obscured by cohort size. The reported hazard and odds ratios, while striking, are based on very few events with wide confidence intervals, and multivariable adjustment in this setting is inherently unstable. This is an interesting concept but claims of independent association and biomarker utility should be tempered pending validation with tissue-level correlation and standardized perioperative sampling.

Version 2:

Reviewer comments:

Reviewer #3

(Remarks to the Author)
I thank the authors for clarifying my concerns and providing a detailed description of sample collection. While sample size remains a concern, this could be handled in future studies.

Open Access This Peer Review File is licensed under a Creative Commons Attribution 4.0 International License, which permits use, sharing, adaptation, distribution and reproduction in any medium or format, as long as you give appropriate credit to the original author(s) and the source, provide a link to the Creative Commons license, and indicate if changes were made.
In cases where reviewers are anonymous, credit should be given to 'Anonymous Referee' and the source.
The images or other third party material in this Peer Review File are included in the article's Creative Commons license, unless indicated otherwise in a credit line to the material. If material is not included in the article's Creative Commons license and your intended use is not permitted by statutory regulation or exceeds the permitted use, you will need to obtain permission directly from the copyright holder.
To view a copy of this license, visit https://creativecommons.org/licenses/by/4.0/

RESPONSE TO REVIEWERS

In summary, since our last submission, we have:

- Characterized the taxa of collagenase-producing strains that resided within the collagenolytic bacterial community associated with recurrence. Further, using matched non-collagenase-producing strains, we have demonstrated that these collagenolytic strains have unique genomes and can promote cancer cell migration and invasion *in vitro* in a collagenase-dependent manner.

- Carefully reviewed the entire manuscript, and the conclusions were tempered throughout to better fit the presented data.

- Addressed all methodological questions that were raised by the reviewers.

Specifically:

Reviewer #1
Thank you for the favorable review of the manuscript.

Reviewer #2

Reviewer Comment 1: Many of the conclusions continue to extend beyond what the data supports. For example, references to a “pro-metastatic collagenolytic microbiota” or statements such as “the in vitro analysis demonstrates that the collagenolytic microbiota…promotes tumor progression” (lines 269–270) are not substantiated. The authors tested uncharacterized supernatants from mixed fecal cultures on 2D cell lines, not defined taxa or in vivo tumors. These experiments can only be interpreted as cell line phenotypic changes, not tumor progression. The repeated use of terms like “pro-metastatic collagenolytic microbiome” should be tempered unless supported by experiments using defined, patient-derived isolates with demonstrated collagenolytic activity.

Author Response: Thank you for this comment, and it was not our intention to make a conclusion beyond what our data supports. We thoroughly reviewed the entire manuscript and tempered the conclusions and verbiage throughout. We hope that the reviewer agrees with the current language.

Reviewer Comment 2: As I noted previously, a central weakness is the reliance on uncharacterized fecal supernatants. Without identifying specific bacterial taxa or quantifying their contributions, the results cannot be independently reproduced or validated. I strongly encourage the authors to sequence protease-positive colonies from their skim-milk agar assays via colony PCR + Sanger sequencing + BLAST to ID followed by WGS (very reasonable now, only $50-65) of isolates of interest and then test defined isolates in functional assays. This would provide a reproducible framework and allow others to confirm these findings and enormously strengthen the manuscript.

Author Response: Thank you for this comment, and you are correct that we should have performed this analysis in the original submission. In this resubmission, we have now identified specific bacterial isolates that promote cancer cell migration and invasion.

To do this, we speciated stool samples collected on the day of surgery from all patients who developed recurrence on non-selective agar plates overlaid with milk. We then picked protease-positive and protease-negative colonies from each patient (10-12 colonies per patient for a total of 72 colonies) to make a bacterial library. Collagenase activity was confirmed by growing each isolate in liquid broth and checking collagenolytic activity using fluorescent gelatin (EnzChek gelatinase/collagenase assay kit). Sanger

sequencing was then used to determine the identity of each isolate in this 72-strain library. Text describing this experimental plan has been added to the methods section (**lines 416-434**).

Results showed that, similar to our previous work, *Enterococcus* genus was the predominant genus within this strain library, and we identified the existence of both collagenase-producing and non-collagenase strains of *E. faecium*, *E. durans*, and *E. avium*. This library is presented in the new **Supplementary Table 2**. To determine whether these individual strains could promote cancer progression, we selected collagenase-producing and non-collagenase-producing strains from each *Enterococcal* species and performed *in vitro* migration and invasion assays. In addition, because it demonstrated the most collagenase activity *in vitro*, we also tested a strain of *Pseudomonas aeruginosa*. As with the entire collagenolytic bacterial community, we found that the supernatant from these collagenolytic bacterial strains significantly increased both migration and invasion of human-derived cancer cells (SW480, HCT116), whereas the non-collagenolytic strains had no effect. This data is now presented within the text (**lines 259-277**) and in the new **Supplementary Figure 5.**

Finally, we performed whole-genome sequencing of these isolates, which confirmed taxonomic identity and purity of a single strain used for the *in vitro* assays. We further inspected the genomic differences between the matched collagenase-producing and non-producing strains. Functional annotation revealed that all collagenase-producing strains harbor a more diverse genome with a larger metabolic capacity, and increased species-specific genes involved in virulence, such as the Type IV secretion system (PF16943), adhesion proteins (PF05738), and staphopain peptidase C47, which breaks down collagen (PF05543). This data is now presented within the text (**lines 279-295**), and new **Supplementary Figures 6** and **7**, **Supplementary File 1**, and the experimental plan has been added to the methods section (**lines 470-480**).

Together, we have now characterized the taxa of collagenase-producing strains that resided within the collagenolytic bacterial community associated with recurrence. Further, using matched non-collagenase-producing strains, we have demonstrated that the collagenolytic strains have unique genomes and can promote cancer cell migration and invasion *in vitro* in a collagenase-dependent manner. We agree with the reviewer that the inclusion of this data greatly strengthens our manuscript and allows other investigators to confirm our findings and/or perform follow-up studies.

Reviewer Comment 3: Bacterial load (Fig 1E): Please clarify whether 16S copy numbers were normalized per gram of stool. Post–bowel prep, the absolute mass of colon contents is drastically reduced, and without normalization these comparisons are misleading.

Author Response: Yes, the 16S copy number was normalized to the mass of the stool, which, as mentioned by the reviewer, is critical for data interpretation. This was mistakenly not described in the methods of the original submission; we have added text to clarify (**lines 161, 439-440**).

Reviewer Comment 4: Collagenase activity (Fig 2B): One NR sample shows measurable activity. Was stool actually obtained, and was bowel prep complete? How was input normalized for the broth assays (e.g., equal CFUs)? Could higher inoculum in NR samples yield activity comparable to R? These clarifications are important to assess whether differences reflect true biology or just variation in input.

Author Response: Thank you for this comment and for allowing us to clarify. All 19 patients had a complete bowel prep and their colon was not filled with stool. In all patients, however, when the specimen was opened in the pathology lab, residual stool was present in all patients and was in contact with the tumor. This stool is the biospecimen that was collected and termed 'day of surgery' sample. We have added text to clarify this (**line 412**).

For the collagenase broth assays (Fig. 2B), the collagenase activity is normalized to bacterial growth via optical density measurement (OD at 600 nm). Given this normalization, we are able to assess collagenolytic

activity per bacterial growth. We have added clarifying text to the methods section (**lines 515-516**) and changed the Y-axis for **Fig. 2B**.

Reviewer Comment 5: Migration/invasion assays (Fig 2E–G): How were supernatants standardized? Without normalization to bacterial load, CFU, or biomass, it is unclear whether the observed effects are due to collagenase or other factors in the mix.

Author Response: Thank you for this comment. Yes, supernatants were normalized and we apologize for the lack of clarity. To make supernatants, bacteria were first revived by placing a loop of glycerol stock aliquots into 2 ml of liquid TSB and incubating the culture at 37°C overnight. To normalize the number of bacteria used to create the supernatant, this culture was then diluted in fresh TSB to obtain an OD (600nm)=0.5. To make cell-free supernatants, the bacterial culture was centrifuged (4,000 rpm, 10 min, 4°C) to separate bacterial cells and the secreted fraction (supernatant), and the supernatant was sterilized using a 0.2 µm filter. For migration and invasion assays, 50 µl of the supernatant was added to cells sitting in the upper chamber of the transwell in 150 µl volume of their own medium (25% of the total volume). Text was added to the methods section for clarification (**lines 484-485, 537**).

Reviewer Comment 6: Lines 229–231 appear contradictory: the text states there was no difference in collagenolytic activity on the day of surgery, yet Fig. 2B suggests otherwise. Please clarify.

Author Response: Thank you for pointing this out. This was a typo and is fixed.

Reviewer Comment 7: The manuscript reports no significant global difference in pre–bowel prep microbiome composition or collagenase activity between recurrence and non-recurrence groups, yet the discussion refers to a "bowel-prep resistant, antibiotic-resistant collagenase bacterial signature." It is unclear where this signature is arising from if it is not evident in the pre–bowel prep samples. In their response to my earlier concern about the timeline to metastasis, the authors acknowledged that they were not suggesting that bacteria present at the time of surgery were driving recurrence. Instead, they now hypothesize that the bowel prep "uncovered" a collagenolytic, pro-tumor bacterial community that must have been present beforehand and interacted with the primary tumor to drive micrometastatic disease prior to surgery. This interpretation seems inconsistent with their own data showing no pre–bowel prep differences and raises questions about whether the "bowel-prep resistant collagenolytic signature" is truly causal, or whether it is a correlate of recurrence identified only after manipulation by the bowel prep itself.

Author Response: This is an important comment, and thank you for bringing this up. It is true that phenotypic differences, such as collagenase expression and the promotion of cancer cell migration and invasion, were observed only in the 'day of surgery' samples, only after bowel preparation. However, we did observe differences prior to bowel prep in both β-diversity PC2 (**Fig. 1B**) and antibiotic resistance, both overall resistance genes (**Fig. 1F**) and genes specific to the antibiotics given for the bowel prep (**Fig. 1G**), between patients who developed recurrence and those who did not. This suggests that, even prior to bowel preparation, bacterial composition and function differ between individuals who later develop recurrence and those who do not. While outside the scope of the current report, we hypothesize that, potentially due to increased antibiotic resistance, the antibiotic bowel prep uncovers a virulent microbial community, of which collagenase is an expressed virulent factor.

Reviewer Comment 8: Given the authors' prior findings on Enterococcus faecalis, was this taxon enriched in recurrence cases pre- or post–bowel prep? This analysis would provide a mechanistic bridge between the current work and earlier studies.

Author Response: We thank the reviewer for this comment and have previously performed these analyses. Inspection of the 16S data did not reveal a significant difference in *Enterococcus* ASV between patients who developed recurrence and those who did not. However, we do not believe this detracts from the *in vitro* isolation findings, as these ASVs were cultured from stool, suggesting that these *Enterococci* were present

in the stool but below the limit of detection at the sequencing depth used. As noted above, nearly half of the cultivatable bacteria from stool were genus *Enterococcus*.

.**Reviewer #3**

Reviewer Comment: In this manuscript, the authors have focused on the role of bowel-prep-resistant collagenolytic bacteria that are predicted to promote post-operative recurrence. Although these are interesting findings, the cohort size is simply too small and other clinical factors are missing that can have an impact on recurrence. Examples of a few are the molecular profiles (genetic mutations) of the tumors, grades of the tumor, number of lymph nodes removed, CEA levels, ctDNA status of the blood. I think the best way to conduct the study is to create a repository and wait until many more patients are analyzed, collecting many more clinical datapoints, and if a pattern is found and enough patients are enrolled, potentially performing either subgroup analyses, preferably utilizing propensity score matching (PSM). This is not to say it is not an important finding. It is certainly worth reporting, but the authors need to state it is as such rather than making a statement that suggests such strong clinical relevance. The findings show an association that may have a clinical impact, depending on much larger studies, and hopefully through the formation of a post-surgical microbial repository.

Author Response: Thank you for your comments, and they are well taken. We also agree that other clinical factors that could have influenced recurrence were not reported and should have been. We have now added the distribution of tumor grade and the number of resected lymph nodes in the text (**lines 99-100**) and to **Table 1**. Univariate analysis found no difference between these two variables in patients who developed a recurrence and those who did not. Furthermore, in the same manner as for neoadjuvant/adjuvant therapy, clinical stage, and pathological stage, we performed two additional exact logistic regression analyses to adjust for tumor grade and the number of resected lymph nodes. These results showed that a significant association between collagenase activity and recurrence remained when adjusted for tumor grade (adjusted OR 26.0; p=0.015) and number of nodes removed (adjusted OR 14.9; p=0.0008). This new analysis has been added to the text (**lines 105-106, 230-232, 235-236**).

Additionally, while full molecular profiling was not part of this study, all patients did exhibit microsatellite stability (MSS). This is now documented within the text (**lines 107-108**).

Finally, we fully agree with the reviewer that our preliminary report is limited by a small sample size and that it should be replicated in a larger prospective clinical trial with a longitudinally collected biorepository. In fact, we (the author, BDS) has an NIH R01 grant under review with that exact trial design. Nevertheless, we did not aim to exaggerate our conclusions and have tempered them throughout the manuscript, including the concluding paragraph (**lines 352-358**).

RESPONSE TO REVIEWERS

In summary, since our last submission, we have:

- Added clarifying text for the "day of surgery" sample collection.
- Further tempered the conclusions and stated potential limitations to better fit the presented data.

Specifically:

Reviewer #2

Thank you for the favorable review of the manuscript.

Reviewer #3

Reviewer Comment 1: I remain concerned about the sampling strategy. In practice, stool collection from a freshly resected colorectal specimen is methodologically challenging and potentially variable, and additional procedural detail would be important. Clarification is needed regarding timing from resection to collection, the exact sampling site relative to the tumor and margins, whether the lumen was irrigated or flushed before sampling, and how manipulation or operative contamination was minimized. The "day of surgery" specimen—residual stool in direct contact with the tumor—sits at the center of the manuscript's conclusions, yet without detailed standardization it is difficult to distinguish a true biological signal from procedural variability. More broadly, because this sample is tumor-adjacent, it remains unclear whether the signature reflects a causal microbial phenotype or tumor-associated selection in biologically aggressive cancers - or whether it confers aggressiveness. The absence of tumor tissue or mucosa-associated microbiome analysis further limits mechanistic inference and leaves the driver-versus-correlate question unresolved.

Author Response: Thank you to the reviewer for this comment, and we agree that these details should have been included in the previous submission. To collect the "day of surgery" specimen, once the specimen was physically resected from the patient, the clinical research coordinator immediately carried it from the operating room to the University of Chicago Pathology Department Human Tissue Resource Center (an approximately five-minute walk). Here, a dedicated Human Tissue Resource Center technician, trained in this specific protocol, performed the following standardized procedure to obtain intraluminal stool: First, the specimen was opened longitudinally, away from the tumor, to avoid contamination. Once the specimen was opened, the residual stool on top of and in contact with the tumor was spooned into a sterile cup using a sterile spatula. Stool that was not in contact with the tumor or on the margins was not collected, and to avoid cross-contamination, the lumen was not flushed or irrigated prior to stool collection. All samples were processed immediately by homogenizing them in lysing matrix D (MP Biomedicals, 6913100). Aliquots of homogenized stool were stored either alone or mixed with 20% glycerol at -80°C until they were used for future experiments. This text has now been added to the methods section (**lines 411-419**).

Reviewer Comment 2: The small sample size (n=19; 6 recurrences) is the dominant limitation and materially constrains interpretability. Signals in Supplementary Table 1—such as tumor location (biofilm potential), recent antibiotic exposure, and stage distribution (two-thirds localized overall vs roughly half among recurrences)—raise the possibility of confounding that is likely obscured by cohort size. The reported hazard and odds ratios, while striking, are based on very few events with wide confidence intervals, and multivariable adjustment in this setting is inherently unstable. This is an interesting concept but claims of independent association and biomarker utility should be tempered pending validation with tissue-level correlation and standardized perioperative sampling.

Author Response: Thank you for this comment; it was never our intention to extend our conclusions beyond what our data represent. We have added clarifying text throughout the manuscript to further temper our conclusions and state potential limitations (**lines 297, 309-310, 356**).
